# Single-cell profiling reveals molecular basis of malignant phenotypes and tumor microenvironments in small bowel adenocarcinomas

**DOI:** 10.1038/s41421-022-00434-x

**Published:** 2022-09-14

**Authors:** Jingwei Yang, Xin Zhou, Ji Dong, Wendong Wang, Yongqu Lu, Yuan Gao, Yu Zhang, Yunuo Mao, Junpeng Gao, Wei Wang, Qingqing Li, Shuai Gao, Lu Wen, Wei Fu, Fuchou Tang

**Affiliations:** 1 School of Life Sciences, Biomedical Pioneering Innovation Center, Department of General Surgery, Third Hospital, Peking University, Beijing, China; 2 Beijing Advanced Innovation Center for Genomics (ICG), Ministry of Education Key Laboratory of Cell Proliferation and Differentiation, Beijing, China; 3 Peking University Third Hospital Cancer Center, Beijing, China; 4 Guangzhou Laboratory, Guangzhou, Guangdong China; 5 Department of Breast Surgery, The First Affiliated Hospital of Zhengzhou University, Zhengzhou, Henan China; 6 Academy for Advanced Interdisciplinary Studies, Peking University, Beijing, China; 7 Peking-Tsinghua Center for Life Sciences, Peking University, Beijing, China; 8 College of Animal Science and Technology, China Agricultural University, Beijing, China

**Keywords:** Cancer microenvironment, Gastrointestinal cancer

## Abstract

Small bowel adenocarcinomas (SBAs) are rare malignant tumors with a high mortality rate, and their molecular characteristics are still largely unexplored. Here we performed single-cell RNA sequencing for tumor samples from 12 SBA patients and predicted drug candidates for SBA. We identified four prevalent subtypes of malignant cells with distinct signatures including cell cycle program, mitochondria program, metabolism program and epithelial–mesenchymal transition (EMT) program. The progression relationships of these four subtypes of malignant cells were also revealed, which started from the cell cycle program, through the mitochondria program and then progressing into either the metabolism program or the EMT program. Importantly, ligand–receptor interaction pairs were found to be specifically enriched in pairs of EMT-program malignant cells and highly exhausted CD8^+^ T cells, suggesting that cancer cell subpopulations with EMT features may contribute most to the exhaustion of T cells. We also showed that the duodenal subtype of SBA exhibited molecular features more similar to gastric cancer whereas jejunal subtype of SBA more similar to colorectal cancer. Especially, we predicted specific drugs for SBA based on differential gene expression signatures between malignant cells and normal epithelial cells of SBA, and verified more potent inhibitory effects of volasertib and tozasertib for SBA cancer cells than conventional drugs of SBA at the same concentration, which provides new clues for treatments of SBA. In summary, our study provides a blueprint of the molecular signatures of both tumor cells and tumor microenvironment cells in SBA and reveals potential targets and drug candidates for its clinical treatments.

## Introduction

Small bowel adenocarcinomas (SBAs) are rare gastrointestinal cancers with unfavorable prognoses. More than half of SBAs originate in the duodenum, while 25%–29% arise in the jejunum, and 10%–13% arise in the ileum^1^. Although the small intestine accounts for > 75% of the length of the entire gastrointestinal tract, small intestinal cancers only account for 2% of all gastrointestinal tract tumors^2^, which indicates unique mechanisms of carcinogenesis in the small intestine. Relative to gastric cancer (GC) or colorectal cancer (CRC), the incidence of SBA is rare, but the intrinsic reasons for this are still not clear^3^. Rapid epithelial cell turnover, fast dietary material passage, low bacterial loads, high lymphoid aggregate levels and a relatively alkaline environment may partially explain the low incidence of SBA^4^. Due to the rarity of pathological studies on SBA, chemotherapy regimens for SBA are usually based on the treatment of CRC as a reference, and specific drugs for SBA are lacking. Recently, immunotherapy-related indicators such as DNA mismatch repair-deficient/microsatellite instability-high (dMMR/MSI-H) status, PD-L1 expression and a high tumor mutational burden (TMB) were reported to appear at higher frequencies in SBA than in other gastrointestinal cancers, and pembrolizumab or nivolumab, with or without ipilimumab, can be used as second-line treatment options for advanced dMMR/MSI-H SBA more widely^5^.

Genomic studies have revealed different characteristics of SBA compared to GC and CRC^6–8^. However, there are few studies on the SBA transcriptome, and more specific markers of SBA are required for its diagnosis and prognosis. Previous genomic studies of SBA were mainly based on bulk tissue sequencing data, which reveal the average features of tumor cells and microenvironment cells in tumor tissues, and the intratumoral heterogeneity and cell type composition of SBAs have not been comprehensively studied^9^^,^^10^.

Here we performed single-cell RNA sequencing (scRNA-seq) for samples of multiple sites from 12 SBA patients. The transcriptomic features of malignant cells were comprehensively analyzed, the intratumoral and microenvironment heterogeneity of SBA were systematically revealed, and inter-regulatory processes were inferred from ligand–receptor interactions. In detail, we identified 4 prevalent subtypes of malignant cells with distinct gene expression signatures including cell cycle program, mitochondria program, metabolism program and epithelial–mesenchymal transition (EMT) program. The progression trajectory of these 4 subtypes of malignant cells started from the cell cycle program, through the mitochondria program and then progressing into the metabolism program or the EMT program. Malignant cells with EMT features were also found to contribute most to the exhaustion of CD8^+^ T cells. Using the transcriptome signatures, we identified that the duodenal subtype of SBA exhibited molecular features more similar to GC whereas jejunal subtype of SBA more similar to CRC, which provides new clues for clinical information of SBA. Finally, drug candidates for the treatment of SBA were also predicted based on differential gene expression signatures between malignant cells and normal epithelial cells. Volasertib and tozasertib were verified more efficient for killing SBA cancer cells than frequently-used clinical drugs of SBA in vitro, which may benefit drug discovery and the clinical treatments of SBA.

## Results

### The cellular landscape of SBA revealed by scRNA-seq analysis

**Fig. 1:**
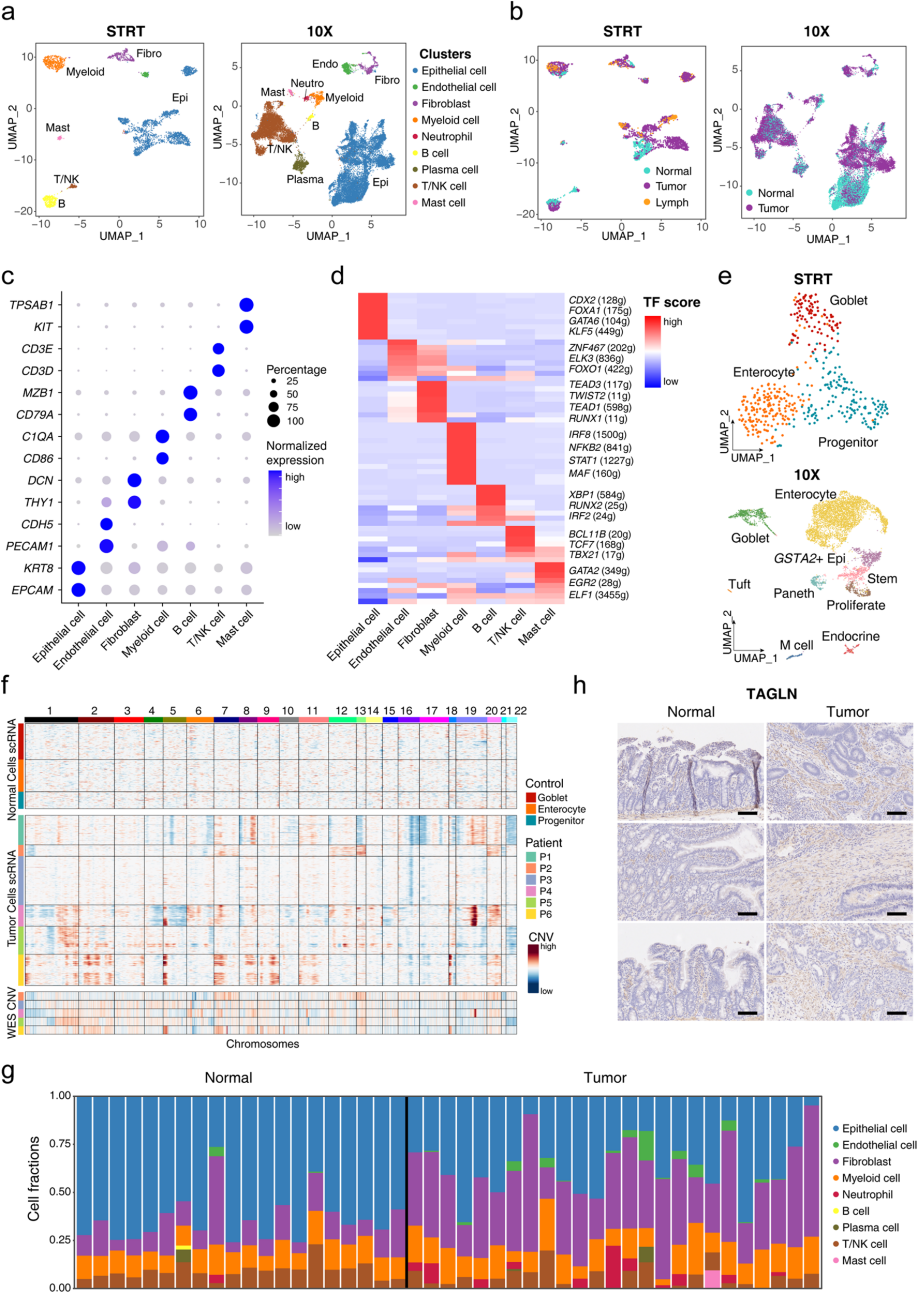
Expression landscape of SBA and cell composition changes. **a** UMAP plot exhibiting the identified clusters of the STRT and 10× datasets. **b** UMAP plot exhibiting the cell tissue sources of the STRT and 10× datasets. **c** Dot plots presenting the normalized expression level of corresponding markers of each cell cluster in the STRT dataset. **d** Heatmap showing the average normalized GRN expression scores. **e** UMAP plot exhibiting the identified clusters of normal epithelial cells in the STRT and 10× datasets. **f** The upper panel exhibiting large-scale CNVs of single cells inferred based on normal epithelial cells in the STRT dataset. The middle panel exhibiting large-scale CNVs of single cells inferred based on epithelial cells from tumor tissues in the STRT dataset. The lower panel exhibiting the CNV called from the WES data of P3–P6. **g** Histogram showing the cell composition percentage of each sample inferred by the deconvolution analysis. **h** Representative IHC staining of TAGLN in adjacent normal and primary tumor tissues (original magnification 100×). The rows of the paired normal and tumor samples are from the same patients, and three individual patients are listed. Scale bar, 100 μm.

To systematically reveal the tumor characteristics of SBA, we collected 34 samples, including 21 primary tumor samples, 10 adjacent normal samples and 3 lymph node metastatic tumor samples from 12 patients of SBA, and performed scRNA-seq analysis for these samples (Supplementary Table S1). We also performed the whole-exome sequencing (WES) for 15 samples from P3–P6, to identify inter-patient and intra-patient genetic heterogeneities of SBA. To equilibrate sequencing accuracy and throughput of the single-cell data, we utilized both the modified high-precision STRT method and the 10× Genomics methods, applying each method in 6 cases, and acquired data from 3676 and 27,390 single cells, respectively, after filtration (Supplementary Fig. S1a)^11,12^. Overall, in both the STRT and 10× Genomics datasets, 7 main cell types were identified according to well-known markers (Fig. 1a–c; Supplementary Fig. S1b–d): epithelial cells, fibroblasts, endothelial cells, myeloid cells, B cells, T/NK cells and mast cells. In addition to these cell types, 2 other specific cell types were found in the 10× dataset: neutrophils and plasma cells derived from B cells. We also performed the gene regulatory network (GRN) analysis and identified the corresponding GRNs for each cell cluster^13^, which included genes such as *CDX2*, *GATA6* and *KLF5* in epithelial cells, *TEAD3* and *TWIST2* in fibroblasts, and *BCL11B*, *TCF7* and *TBX21* in T/NK cells (Fig. 1d; Supplementary Fig. S1e). For normal epithelial cells in the STRT dataset, the epithelial cells from adjacent normal tissues could be divided into three clusters after removing batch effects: progenitor epithelial cells, goblet cells and enterocytes (Fig. 1e; Supplementary Fig. S1f). In the 10× dataset, normal epithelial cells were clustered into nine types including progenitor epithelial cells, stem cells, *GSTA2*^+^ epithelial cells, goblet cells, enterocytes, endocrine cells, Paneth cells, tuft cells and M cells (Fig. 1e; Supplementary Fig. S1g). To evaluate the differences of the STRT and 10× Genomics methods, we integrated all the acquired cells and epithelial cells from these two datasets (Supplementary Fig. S2e, f). Epithelial cells, stromal cells and immune cells from the STRT and 10× datasets were properly integrated together (Supplementary Fig. S2g). Subclusters of epithelial cells from these two datasets were also integrated well. The same cell types including progenitor cells, goblet cells and enterocytes from both datasets were clustered together or closely, indicating that there were no dramatic differences between these two technical platforms (Supplementary Fig. S2h). The inference of copy number variations (CNVs) by scRNA-seq data was performed, and the normal epithelial cells were used as reference cells with no prominent CNVs, which was confirmed by the WES analysis of the same patient (Supplementary Fig. S3e). The CNV patterns of the malignant epithelial cells were found to be heterogeneous among different patients, and some common CNVs shared by different patients were also identified such as gains of Chr5p and Chr7p (Fig. 1f; Supplementary Fig. S2a–c). The inferred CNVs by scRNA-seq data were also verified by calling CNVs from the WES data. We selected out epithelial cells with high CNV levels as malignant cells, and these cells were clustered separately according to the individual patients (Supplementary Fig. S2d).

To assess the changing ratio of identified cell types during SBA tumorigenesis, we identified the cell proportions of different cell types, in 17 tumor samples and 8 adjacent normal tissue samples from 10 patients in which cells are sampled relatively randomly and not enriched for specific cell types (excluding P5 and P6). However, there were essentially no significant differences in cell type fractions between tumor tissues and adjacent normal tissues, probably due to the limited sample number (Supplementary Fig. S3a, b). To compute the fractions of different cell types more accurately, we performed deconvolution analysis of published bulk sample gene expression profiling data of SBA based on the signatures of reference cell types from the 10× dataset^9,14^ (Fig. 1g; Supplementary Fig. S3c). We could clearly see that the percentages of fibroblasts were much higher in the tumor tissues than in the adjacent normal tissues, indicating that fibroblasts are enriched during SBA tumor progression. The decreasing percentages of epithelial cells and increasing percentages of endothelial cells in tumor tissues were also identified. Moreover, the changing tendencies of cell proportions for different cell types were consistent between our single-cell dataset and the deconvolution analysis of bulk sample dataset. TAGLN is a well-known marker of fibroblasts, and the immunohistochemistry (IHC) analysis of TAGLN also verified the increasing proportion of fibroblasts in tumor tissues relative to adjacent normal tissues (Fig. 1h).

As for the WES data, 15 tumor samples from 4 cases were collected to detect somatic mutations. As for somatic mutations in frequently mutated genes, we detected somatic mutations in *TP53*, *SMAD4*, *PIK3CA*, *SOX9*, *ARID2* and *OBSCN* (Supplementary Fig. S3d). Of them, somatic mutations of *TP53* were found in all samples from P4 and P6 and the P3_T3 from P3, somatic mutations of *OBSCN* were found in P4 and P5, somatic mutations of *SMAD4* were found in P4 and P6, and somatic mutations of *SOX9*, *ARID2*, *PIK3CA* were found only in P3, which reveals the inter-patient heterogeneity among patients partially. Mutations of *ERBB2*, *APC*, *KRAS*, *IDH1* and *PTEN* were not detected, which may be due to the limited sample number and their relatively low mutation rate among SBA patients.

### Molecular characteristics of cancer cells in SBA associated with their malignant and pathological phenotypes

**Fig. 2:**
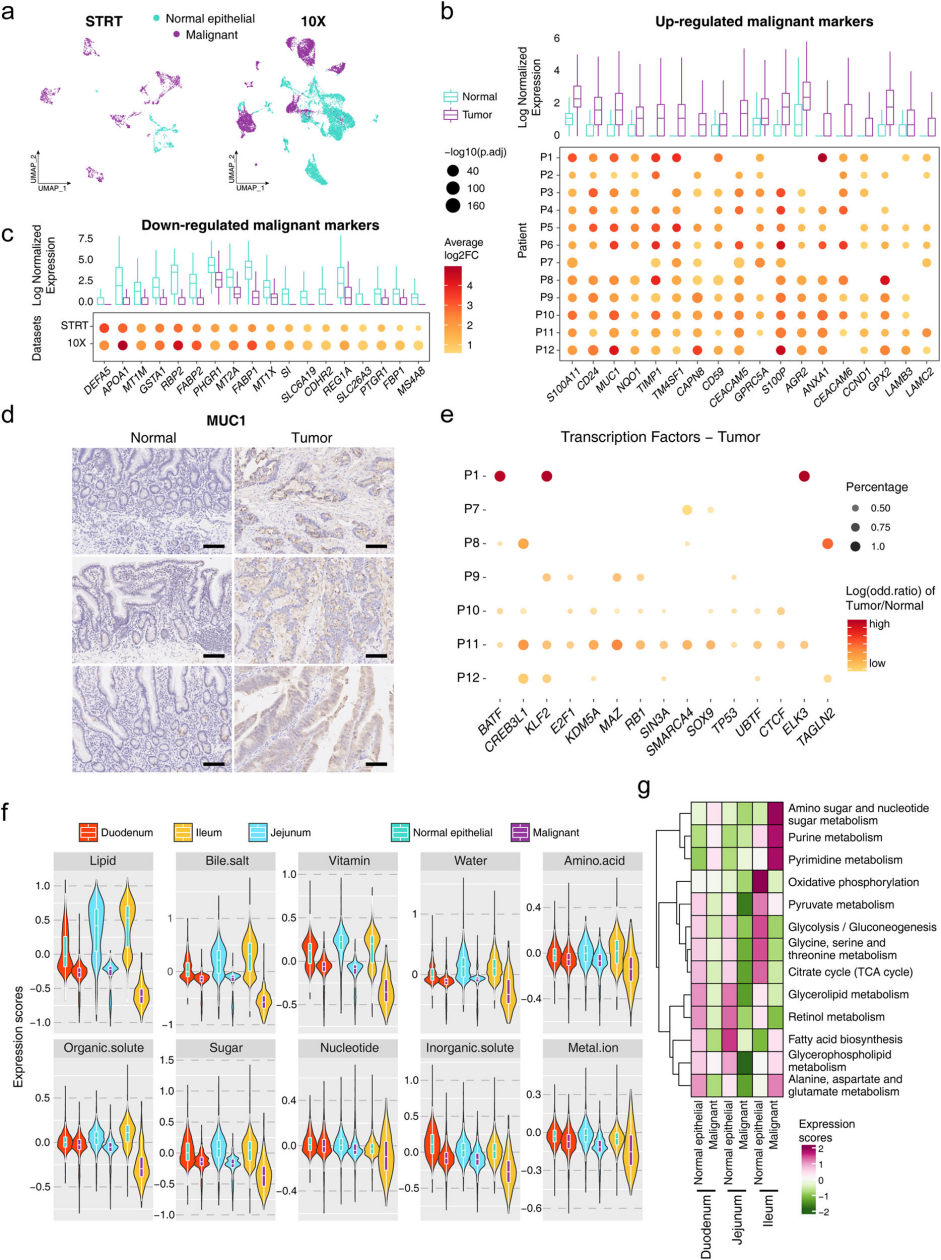
Molecular signatures of the malignant cells and association with pathological characteristics in SBA. **a** UMAP plot exhibiting the cell identification by CNV values in the STRT and 10× datasets. **b**, **c** Boxplots in the upper panel showing average normalized expression of the malignant cells and normal epithelial cells. Dot plots in the lower panel showing the log_2_ fold change (FC) of malignant cell relative to normal epithelial cell of each patient or dataset. **d** Representative IHC staining of MUC1 in adjacent normal and primary tumor tissues (original magnification 100×). The rows of the paired normal and tumor samples are from the same patients, and three individual patients are listed. Scale bar, 100 μm. **e** Dot plot showing the log odd ratio values of tumor/normal of corresponding TFs, with *P* values calculated by χ^2^ test. **f** Violin plot showing the expression scores of absorptive and transport gene sets in malignant and normal epithelial cells from different intestinal regions. **g** Heatmap showing the expression scores of metabolism pathways in malignant and normal epithelial cells from different intestinal regions.

There have been limited gene expression profiling studies of SBA, and the detailed molecular characteristics of malignant cells in SBA are not clear. We defined clusters whose epithelial cells with high CNV levels were dominant as malignant cells (cancer cells), and the other epithelial cell clusters as normal epithelial cells (Fig. 2a). Despite extremely high tumor heterogeneity, malignant cell markers such as *MUC1*, *CEACAM5*, *S100A11*, *CD24*, and *TM4SF1* were relatively uniformly upregulated in cancer cells and had the potential to serve as targets for SBA diagnosis and treatment (Fig. 2b, c; Supplementary Fig. S4a–c, Tables S2–S5). Then, we performed IHC to verify the differential expression of MUC1 between tumor tissues and adjacent normal tissues at the protein level (Fig. 2d). The differential expression in GRNs identified in each patient also confirmed the tumor heterogeneity of SBA, with transcription factors (TFs) such as *BATF*, *CREB3L1*, *KLF2*, *E2F1* variably expressed among different patients (Fig. 2e; Supplementary Fig. S4d). We also compared differentially expressed genes (DEGs) of malignant cells between primary and metastatic tumor samples (Supplementary Fig. S4e). We found that the mesenchymal signature is enriched in the malignant cells from lymph nodes compared to the malignant cells from primary tumors, which may be associated with the enhanced EMT process during metastasis.

To further investigate any residual small intestinal functions of the malignant cells under pathological conditions, we integrated our datasets with another published single-cell dataset from normal ileum samples to assess normal nutrient transportation and absorption functions of intestine epithelial cells for various nutrients (Supplementary Fig. S5a, b)^15^. We selected all the normal epithelial cells from these datasets and evaluated the corresponding score of each gene set. In terms of absorption and transport functions related to lipids, bile salts, vitamins, water, amino acids, organic solutes, sugar, nucleotides, inorganic solutes and metal ions, the malignant cells from the duodenum, jejunum, and ileum showed declined functions relative to normal intestinal epithelial cells, and this trend was consistent throughout the small intestine (Fig. 2f). Especially in functions related to lipids, bile salts, vitamins and sugar, the gene set score of these functions decreased significantly in malignant cells compared to normal epithelial cells in ileum (Supplementary Fig. S5c). Furthermore, we evaluated scores of different metabolic pathways, and found that many metabolic pathways were depleted in malignant cells compared to normal epithelial cells (Fig. 2g). However, several metabolic functions such as amino sugar and nucleotide sugar metabolism, purine metabolism and pyrimidine metabolism, were enriched in the malignant cells of ileum.

### Identification of two tumor progression paths and EMT expression programs that may favor metastasis

**Fig. 3:**
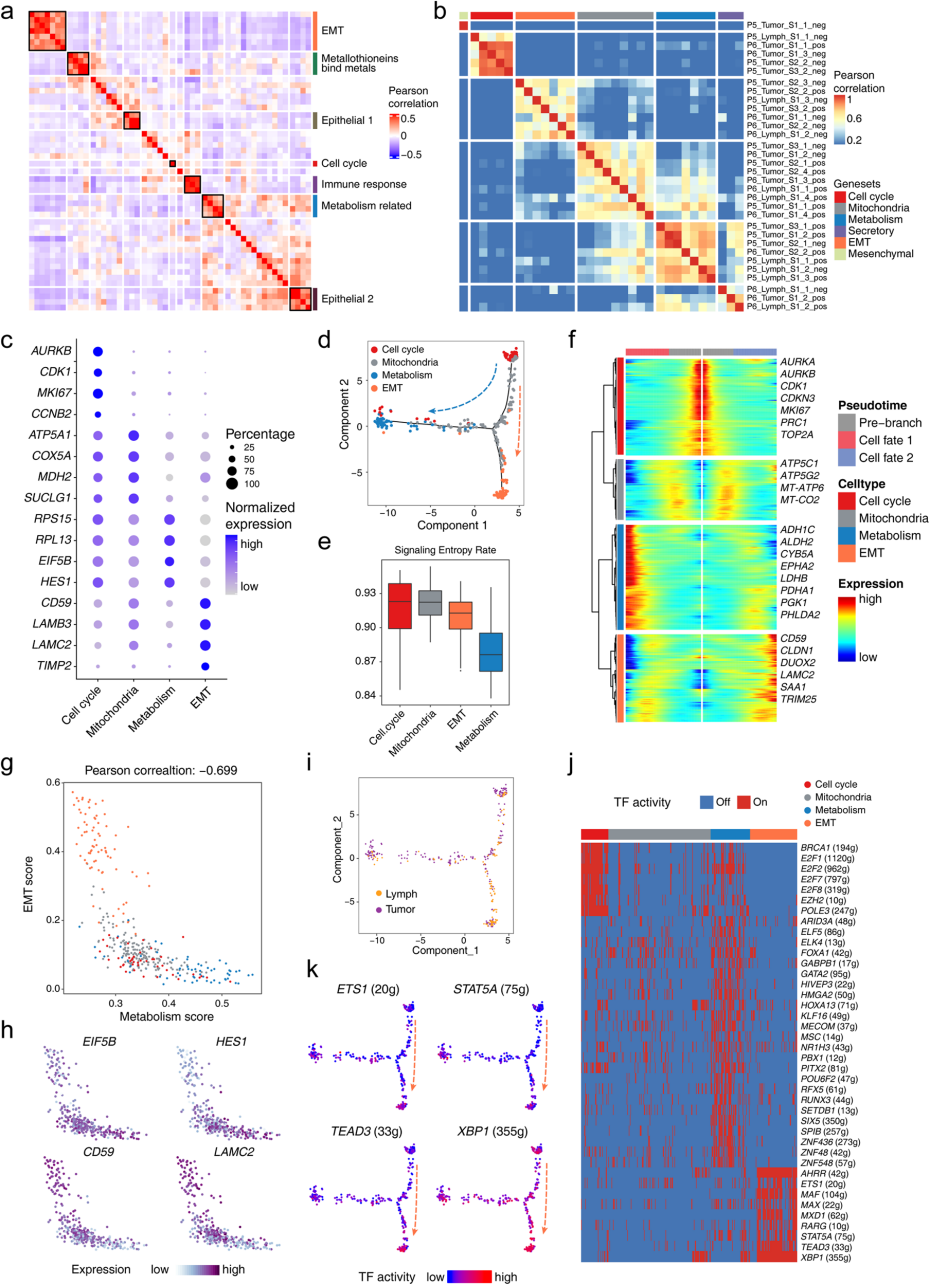
Tumor heterogeneity and gene expression programs of the malignant cells. **a** Heatmap showing the Pearson correlation clustering of identified intra-tumor expression programs. **b** Heatmap showing the Pearson correlation clustering across gene signatures extracted from the PCA output. **c** Dot plots presenting the normalized expression levels of corresponding markers of each gene expression program of the malignant cells in the STRT dataset. **d** Scatterplot showing the developmental trajectory of malignant cell identified by different expression programs using monocle2. **e** Boxplot showing the signaling entropy rate of cells with identified gene expression programs. **f** Heatmap showing diverse expression patterns of the malignant cells from different expression programs along the pseudotime trajectory. **g** Scatterplot exhibiting the negative correlation of metabolism- and EMT-related malignant cells. **h** Scatterplot exhibiting the expression levels of representative marker genes. **i** Scatterplot exhibiting the information of malignant cells from lymph nodes and primary tumor tissues embedded in the trajectory pathway. **j** Heatmap showing the on or off activities in the malignant cells of diverse expression programs. **k** Scatterplot showing the TF expression scores in the developmental trajectory.

After the intrinsic epithelial cell subpopulations and potential sources of malignant cells were identified, the nonnegative matrix factorization (NMF) method was applied to elucidate feasible expression programs with co-expressed genes. Based on all of the malignant cells in the STRT dataset, we generated 50-dimensional principal components and extracted 7 co-expressing programs (Fig. 3a; Supplementary Fig. S6a). The enriched signaling pathways that constituted different expression programs were functionally associated to some extent, especially the epithelium-related signaling pathways.

To further illustrate the detailed characteristics of the malignant SBA cells, we focused on the malignant cells of P5 and P6, who were patients with duodenal cancers with metastatic lymph node tumors. For the tumor sampling sites of P5 and P6, the negative and positive poles of the top principal components obtained from principal component analysis (PCA) were taken (Supplementary Fig. S6b). We scored these features and extracted six programs by hierarchical clustering (Fig. 3b, c). Four gene expression programs, including cell cycle-related program, mitochondria-related program, metabolism-related program and EMT-related program, were common programs shared by these two patients, and the other two programs were each specific to one of the patients (Supplementary Fig. S6c, d, Table S6).

The correlations and progression paths of these four expression programs were analyzed further. The metabolism- and EMT-related programs showed the lowest correlation with each other, which indicated mutually exclusive states (Supplementary Fig. S6e). The pseudotime trajectories of these four programs also presented the similar pattern of the progression tracks (Fig. 3d). Furthermore, we performed the signaling entropy rate analysis of these cell types^16^, identifying that malignant cells of the cell cycle program and mitochondria-related program had higher ‘differentiation’ potency while malignant cells of the EMT- and metabolism-related signatures had lower ‘differentiation’ potency (Fig. 3e; Supplementary Fig. S6f). Therefore, we inferred that the cell cycle program represented the starting state during tumorigenesis (Fig. 3f). The mitochondria-related program clearly represented the intermediate state of the malignant cells, which connected to the other programs. There were two branches of the malignant cell progression trajectory, in which the metabolism-related program and the EMT-related program represented the end states of tumorigenesis. The EMT-program malignant cells and the metabolism-program malignant cells showed a negative correlation of gene expression, with high expression of *CD59* and *LAMC2* in EMT-program malignant cells and high expression of *EIF5B* and *HES1* in metabolism-program malignant cells (Fig. 3g, h). As for the lymph node metastatic malignant cells, these cells are enriched in the EMT program signatures, accounting for 60% in the EMT subtype and 25% in the metabolism subtype (Fig. 3i). It indicated that the EMT program may be involved in the metastatic process. When the analysis was expanded to other cancer cases, we found similar progression paths of the malignant cells in both the STRT and 10× datasets, except in P1 due to prior chemotherapy (Supplementary Fig. S6g).

We subsequently focused on the EMT program. Among the identified EMT program markers, *LAMB3* and *LAMC2* have also been reported as markers of a non-typical EMT program in head and neck cancers^17^. We also identified related GRNs in the EMT-related program, which included genes such as *ETS1*, *STAT5A*, *TEAD3* and *XBP1* (Fig. 3j, k). Among these genes, previous studies showed that the TF *ETS1* can enhance the expression of *ZEB1/2*, whereas *STAT5A* can induce EMT through *TWIST1*^18,19^. *XBP1* can induce EMT by promoting the expression of *SNAI1/2* in the IRE1-XBP1-snail pathway^20^. These key TFs may be upstream factors promoting EMT. Therefore, we inferred that the identified EMT signature is the initial stage of the typical EMT program, through inducing activities of related TFs to promote EMT.

### Changes in the features of cancer-associated fibroblasts during tumor progression

**Fig. 4:**
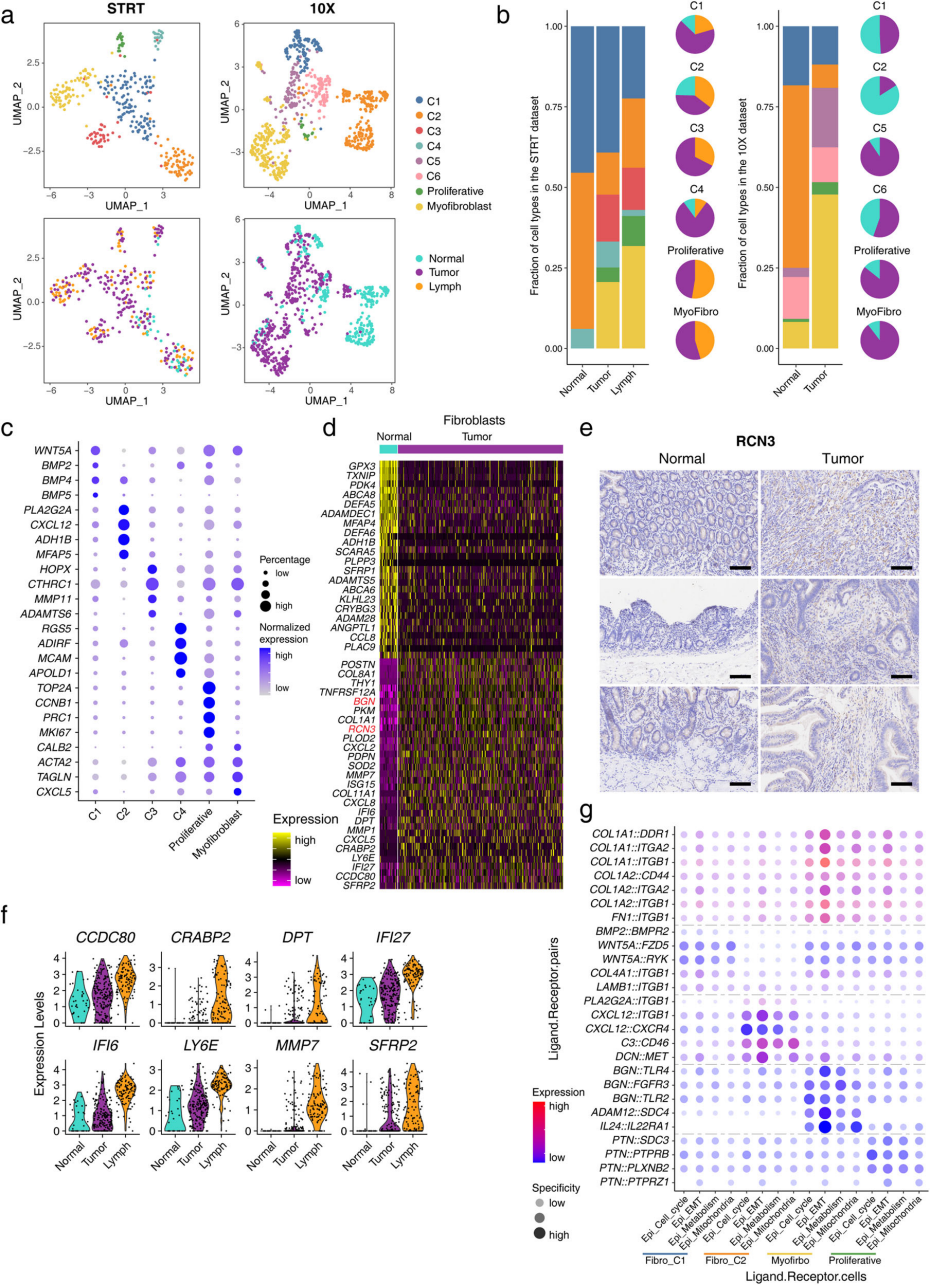
Changes in features of fibroblasts during tumor progression. **a** UMAP plot exhibiting the identified clusters and sources of fibroblast in the STRT and 10× datasets. **b** Histogram showing cell type percentage of fibroblasts from different sources in the STRT and 10× datasets. Pie plot showing fibroblast source percentages in different cell types. **c** Dot plots presenting the normalized expression levels of corresponding markers of each fibroblast cluster in the STRT dataset. **d** Heatmap showing expression of marker genes of fibroblasts from tumor and normal tissues in the STRT dataset. **e** Representative IHC staining of RCN3 in adjacent normal and primary tumor tissues (original magnification 100×). The rows of the paired normal and tumor samples are from the same patients, and three individual patients are listed. Scale bar, 100 μm. **f** Violin plot exhibiting expression of continuously increasing markers during tumor progression in the STRT dataset. **g** Dot plot showing expression of interaction pairs in fibroblast and epithelial cell pairs from different subclusters.

To reveal the characteristics and heterogeneities of cancer-associated fibroblasts (CAFs) in SBA, we enriched fibroblasts from 2 patients in the STRT dataset (Supplementary Table S1). We captured 339 fibroblasts from the STRT dataset and 787 fibroblasts from the 10× dataset (Fig. 4a, c; Supplementary Fig. S7a). After removing batch effects, 6 clusters of fibroblasts were identified in each of the datasets, and 4 clusters were shared by these two datasets (C1, C2, proliferative fibroblasts and myofibroblasts). According to both the STRT and 10× datasets, the percentage of C1 clusters decreased in tumor tissues relative to adjacent normal tissues, while the percentages of proliferative fibroblasts and myofibroblasts increased (Fig. 4b). *ACTA2* is one of the markers for myofibroblasts, and the IHC staining of ACTA2 confirmed that the proportion of myofibroblasts is higher in tumor tissues compared to adjacent normal tissues (Supplementary Fig. S7b).

Next, we explored the alterations of fibroblasts during tumor progression in SBA. Several of the identified DEGs, such as *RCN3*, *BGN*, *TPM4*, and *PKM*, have been reported as marker genes of CAFs in CRC and indicated similar signatures of gastrointestinal cancers (Fig. 4d; Supplementary Fig. S7c, d)^21^. The IHC staining also showed the higher expression of RCN3 in tumor tissues than in adjacent normal tissues, which confirms the upregulation of *RCN3* in fibroblasts during tumorigenesis of SBA (Fig. 4e). Moreover, in the STRT dataset, we identified marker genes of fibroblasts from lymph node tumor sites and 8 genes whose expression levels continuously increased during tumor progression and metastasis (Fig. 4f; Supplementary Fig. S7e). *DPT* and *SFRP2* were rarely expressed in normal epithelial cells and tumor immuno-microenvironment cells but were mainly expressed in fibroblasts from tumor tissues.

Then, we focused on cell communications between fibroblasts and malignant cells. We applied NATMI to quantify the expression and specificity of specific interaction pairs and filtered the ligand–receptor pairs to retain significant interaction pairs among different states (Supplementary Fig. S8a, b)^22^. Eighteen continuously upregulated and nine continuously downregulated differentially expressed interaction pairs from normal tissues to primary tumors and then to lymph node metastatic tissues were validated. Next, we analyzed the ligand–receptor interactions between diverse subtypes of fibroblasts and malignant cells characterized by the identified gene expression programs. We identified interaction pairs in both the STRT and 10× datasets (Supplementary Fig. S8c). The collagen–integrin interaction pairs of myofibroblasts and EMT-malignant cells showed the strongest and most specific interactions among all the identified cell type interactions (Fig. 4g). At the same time, we found that collagen-related genes were more enriched in tumor tissues than in the adjacent normal tissues, which was confirmed by the gene set enrichment analysis (GSEA) of the collagen-related and collagen formation datasets (Supplementary Fig. S8d).

### CD8^+^ T cells with exhaustion signatures and their potential interactions with the malignant cells

**Fig. 5:**
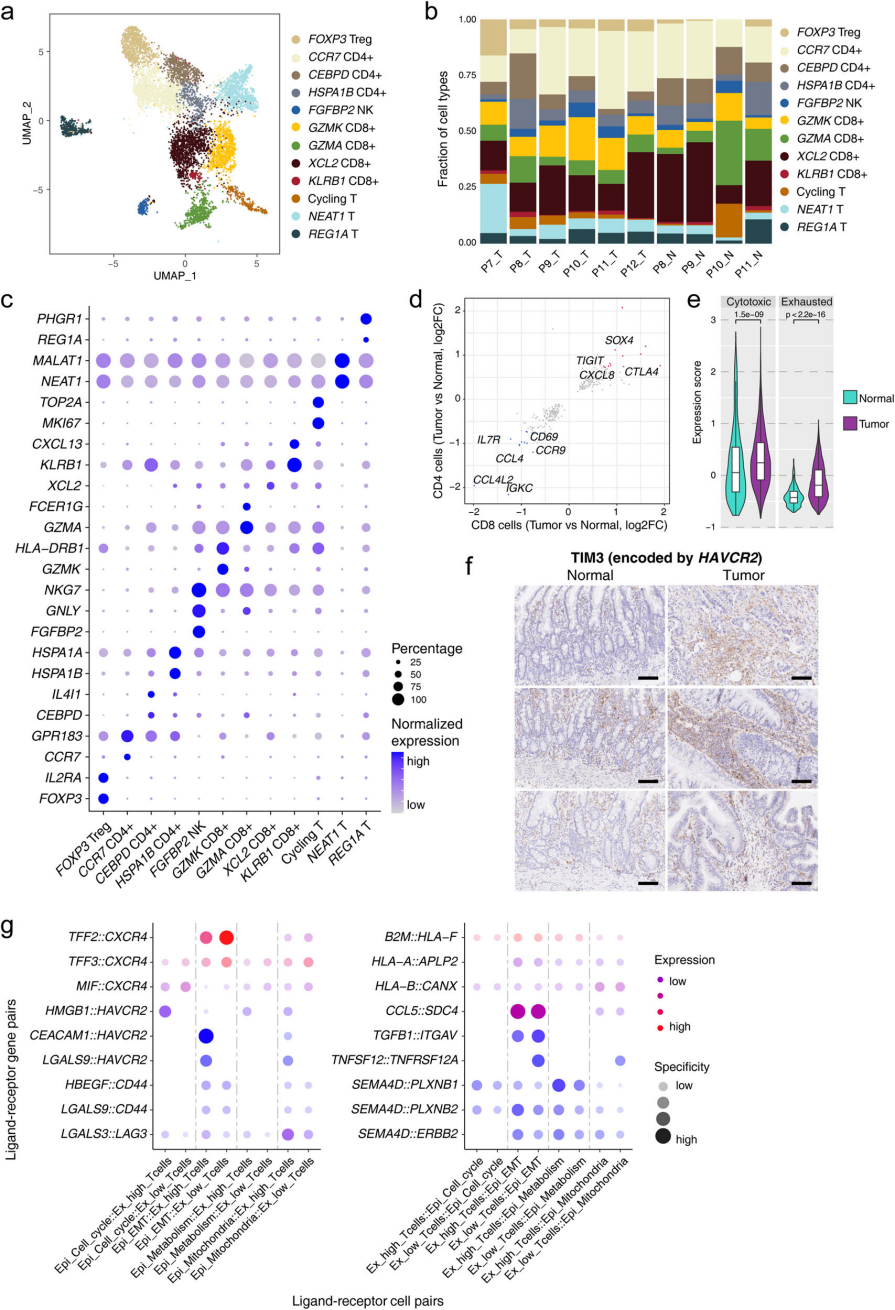
CD8^+^ T cells with exhaustion signatures and related interactions with malignant cells. **a** UMAP plot exhibiting the identified clusters of T cells in the 10× dataset. **b** Histogram showing cell type percentages of different T cells from collected samples in the 10× dataset. **c** Dot plots presenting the normalized expression levels of corresponding markers of T cell clusters in the 10× dataset. **d** Scatterplot showing DEGs of T cells from tumor tissues compared with normal tissues in both CD4^+^ and CD8^+^ T cells. **e** Violin plots showing expression of the exhaustion and cytotoxicity gene sets in CD8^+^ T cells from tumor and normal tissues. The *P* value was calculated by *t*-test. **f** Representative IHC staining of TIM3 in adjacent normal and primary tumor tissues (original magnification 100×). The rows of the paired normal and tumor samples are from the same patients, and three individual patients are listed. Scale bar, 100 μm. **g** Dot plot showing the expression of interaction pairs in epithelial cell and T cell pairs with different signatures.

In our datasets, we captured a sufficient number (7742) of T/NK cells from the 10× dataset and performed downstream analysis. Twelve clusters of T/NK cells were identified, which included one cluster of NK cell, four clusters of CD8^+^ T cells, four clusters of CD4^+^ T cells and three clusters of T cells with other representative features (Fig. 5a, c; Supplementary Fig. S9a). Among these clusters, the cell type composition of T cells from individual samples differed substantially (Fig. 5b; Supplementary Fig. S9b). In general, the percentages of *FOXP3* Treg cells increased significantly in tumor tissues, which indicated a general suppression of T cell activities in SBA. Next, we compared the gene expression profiles of tumor tissue-derived and normal tissue-derived T cells. Taking CD4^+^ Treg cells and CD8^+^ cytotoxic T cells as examples, Treg cells derived from tumor tissues showed higher expression of *LAYN*, *LGALS1* and *TNFRSF1B*, and cytotoxic T cells derived from tumor tissues showed higher expression of *TNFRSF9* (encoding 4-1BB), *LAYN* and *CTLA4* (Supplementary Fig. S9c). We identified DEGs in both CD4^+^ and CD8^+^ T cells from tumor tissues relative to those from adjacent normal tissues (Fig. 5d). As expected, DEGs that were upregulated in T cells from tumor tissues, such as *CTLA4* and *TIGIT*, were enriched for exhausted T cell functions. Downregulated DEGs such as *IL7R*, *CD69*, *CCL4*, *CCL4L2* were associated with T cell activation and cytotoxic activities^23,24^.

Then, we focused on the association of CD8^+^ T cells and exhaustion status. Using the cytotoxicity and exhaustion gene sets, we showed that the T cells from tumor tissues presented significantly higher expression of both the cytotoxicity and exhaustion signature genes than T cells from normal tissues (Fig. 5e). On the basis of the exhaustion gene set, these CD8^+^ T cells were divided into ex-high (highly exhausted signature) and ex-low (lowly exhausted signature) groups (Supplementary Fig. S9d). As expected, the ex-high group showed higher expression of exhaustion signature genes, and the lowly exhausted group presented more T cell cytotoxicity- and leukocyte migration-related signatures (Supplementary Fig. S9e). Among different types of CD8^+^ T cells, exhaustion markers such as *TIGIT*, *BATF*, *CTLA4*, *LAYN* and *PDCD1* were enriched in *KLRB1*^+^CD8^+^ T cells, indicating that this cluster of CD8^+^ T cells contributes most to T cell exhaustion (Supplementary Fig. S9f). *KLRB1*^+^CD8^+^ T cells also highly expressed *CXCL13* (Supplementary Fig. S9g). Of these markers, *KLRB1* was reported to inhibit key aspects of cytotoxic T cell functions significantly in gliomas^25,26^. *CXCL13* has also previously been reported to be associated with exhaustion signatures^27,28^.

Finally, we performed the cell–cell communication analysis of ex-high and ex-low T cells with malignant cells (Fig. 5g). Among these interactions, EMT-program malignant cells and mitochondria-program malignant cells were the most frequent malignant cell types interacting with T cells. Among the interaction pairs of malignant cells and T cells, the T cell receptor *CXCR4* was identified in many interactions, and *CXCR4*-related interactions were enriched in ex-low T cells. We also identified T cell exhaustion-associated interactions among these cell pairs, and as expected, these interactions (such as *HMGB1–HAVCR2*, *CEACAM1–HAVCR2*, *HBEGF–CD44* and *LGALS3–LAG3*) were enriched in ex-high T cells, as receptor-providing cells. *HAVCR2* (encoding TIM3) was one of the main receptors of ex-high T cells, and the IHC results verified that the expression of TIM3 at the protein level was clearly higher in tumor tissues compared with adjacent normal tissues (Fig. 5f). Exhaustion ligands (*CEACAM1*, *LGALS3*, *LGALS9*, etc.) were mainly enriched in EMT-program and mitochondria-program malignant cells, which corresponded to the more malignant cells in our study. We also used the EMT and highly exhausted CD8^+^ T cell gene sets to explore the relationship with highly exhausted T cell interactions in the 10× datasets of GC and CRC^29,30^. We isolated malignant cells and T cells from these datasets and divided them into cells with high or low expression of the EMT or exhausted T cell signature (Supplementary Fig. S10a, b). Although the exhaustion interactions were mainly enriched in highly exhausted T cells, there were no significant differences between low- and high-EMT malignant cells for the interactions (Supplementary Fig. S9h). This suggests that the EMT-high malignant cells promote CD8^+^ T cell exhaustion through cell–cell communications specifically in SBA, but not in GC or CRC.

### Identification of transcriptomic characteristics and relationships of SBA with other gastrointestinal cancers

**Fig. 6:**
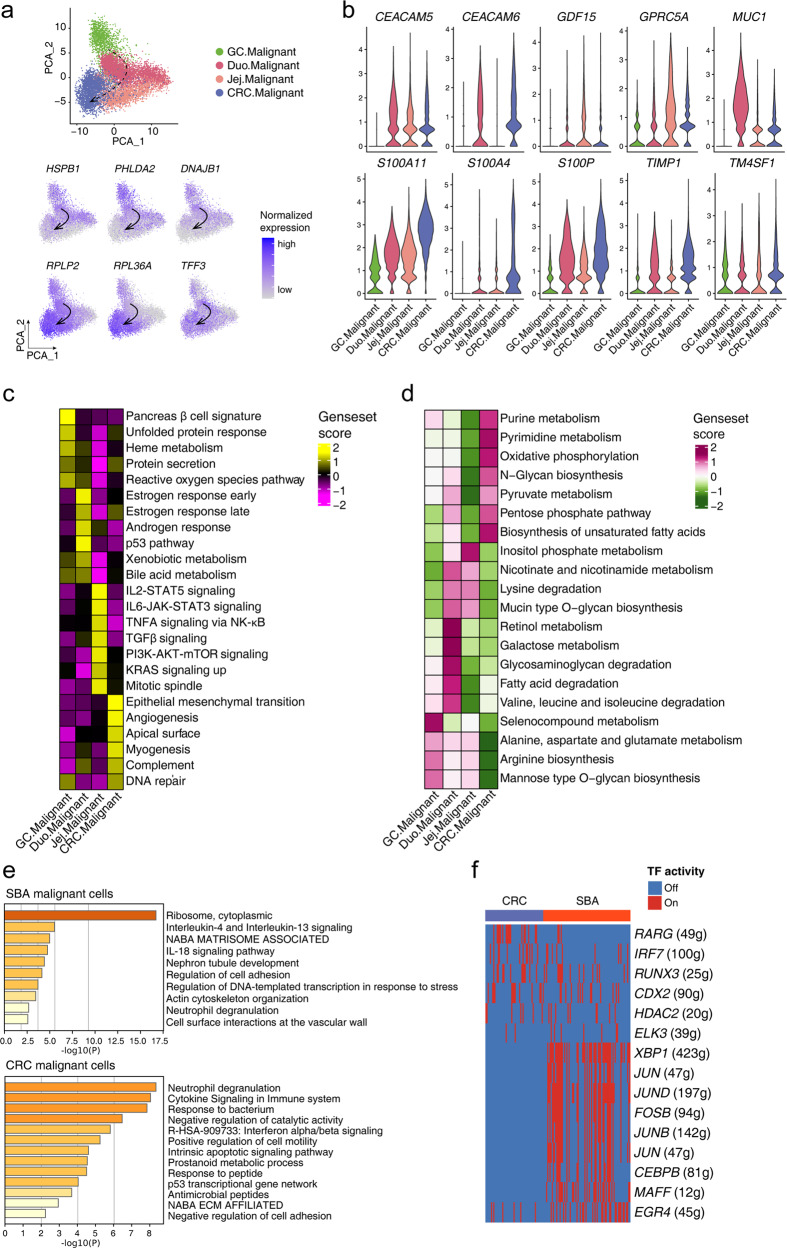
Identification of the similarity and differences of SBA with other gastrointestinal cancers. **a** Scatterplots exhibiting the PCA dimension reduction analysis for gastric, duodenal, jejunal to colorectal malignant cells and DEGs expressed in the PCA plot. **b** Violin plot exhibiting markers of SBA expressed in gastrointestinal cancers. **c**, **d** Heatmap showing gene set scores of hallmark gene sets and metabolism pathways in malignant cells from different gastrointestinal regions. **e** Enrichment analysis for markers of malignant cells in SBA and CRC. **f** Heatmap showing the TF activities of GRNs in malignant cells from SBA and CRC.

The SBA therapies applied to date have mainly mirrored those for CRC, but several studies have suggested that there are significant differences in mutation signatures and genome-wide CNVs between SBA and CRC^6–8^. To reveal the similarities and differences of different gastrointestinal cancers, we identified the correlational relationships in gastrointestinal cancers through integrating our 10× dataset with other published 10× single-cell datasets of gastrointestinal cancers^29,30^, including SBA, GC and CRC (Supplementary Fig. S10a, b). We utilized the features of inferred CNVs to divide epithelial cells into malignant cells and normal epithelial cells in separate datasets with the previously mentioned method, while there were no samples from ileum in our 10× dataset and we divided SBAs in the 10× dataset into duodenal and jejunal subtypes to reveal detailed positional information. Based on the expression of common highly variable genes (HVGs), we scored the correlations among these gastrointestinal cancers (Supplementary Fig. S10c). Although there were no ileal samples in the 10× datasets of SBA, we clearly observed a closer relationship of duodenal subtypes with GC than with CRC. The correlational relationships of gastrointestinal cancers corresponded to the spatial order of the gastrointestinal tract, providing a continuously changing signature across gastrointestinal cancers. To identify the relationship of these gastrointestinal cancers further, we performed the PCA dimension reduction analysis based on the DEGs of gastric and colorectal malignant cells. The PCA analysis showed that the duodenal subtype of SBA exhibited molecular features more similar to GC whereas jejunal subtype of SBA more similar to CRC (Fig. 6a). There is also a trajectory in accordance with the spatial order of the gastrointestinal tract, which is from gastric, duodenal, jejunal to colorectal malignant cells. We identified DEGs of malignant cells from GC and CRC (Supplementary Fig. S10d), and tracked these DEGs in the identified trajectory in the PCA plot. We found that some upregulated DEGs in GC malignant cells such as *HSPB1*, *PHLDA2*, *DNAJB1* have the decreasing expression tendency in malignant cells from GC, duodenal caners, jejunal cancers to CRC. Meanwhile, several upregulated genes in CRC malignant cells such as *RPLP2*, *RPL36A*, *TFF3* have the increasing expression tendency in malignant cells from GC, duodenal cancers, jejunal cancers to CRC (Fig. 6a). The analysis based on scRNA-seq data was consistent with conclusions based on the comparison taking malignant cell as a whole, which indicates the consistency of the identified potential ordered relationships between GC and duodenal SBA and between jejunal SBA and CRC.

To assess the similarities and differences, we integrated these datasets and found that markers of malignant cells in SBA identified previously showed widespread expression among all gastrointestinal cancers (Supplementary Fig. S10e), although some biomarkers, such as *GDF15*, *MUC1*, and *S100A4*, were expressed only in specific intestinal regions (Fig. 6b). We also compared the differentially expressed pathways and metabolic activities among these cancer types (Fig. 6c, d). Secretory-related pathways (pancreatic beta cell and protein secretion signatures) were enriched in malignant cells of GC, hormone response signatures (androgen and estrogen responses) were specifically enriched in duodenal malignant cells, jejunal malignant cell presented proliferation-related signatures (TGFβ and mitotic spindle signatures), while colorectal malignant cells tended to show stronger metastatic characteristics (EMT and angiogenesis signatures). As for metabolic activities, retinol metabolism and galactose metabolism were enriched in duodenal malignant cells, while pyruvate-related metabolism, such as purine and pyruvate metabolism, and biosynthesis-related pathways, such as pentose phosphate pathway and biosynthesis of unsaturated fatty acids, were enriched in colorectal malignant cells.

Differences of SBA and CRC are meaningful to guide treatments of SBA. Among the DEGs found between malignant and normal epithelial cells in SBA and CRC, there were only a few overlaps (Supplementary Fig. S10f). The GO analysis indicated that both malignant cells of SBA and CRC presented EMT signatures (Fig. 6e). The hallmark EMT gene set had a higher expression in CRC than in SBA at the global transcriptome level (Supplementary Fig. S10g). To further distinguish the EMT programs of SBA and CRC, we identified the specific transcriptional activity of *XBP1* in SBA (Fig. 6f). The transcriptional activity of *XBP1* in SBA seems higher than that in CRC. The molecular classifications of SBA and CRC are complex, and more studies are needed to identify the EMT signatures in these two cancer types.

### Evaluation of potential targets for SBA

**Fig. 7:**
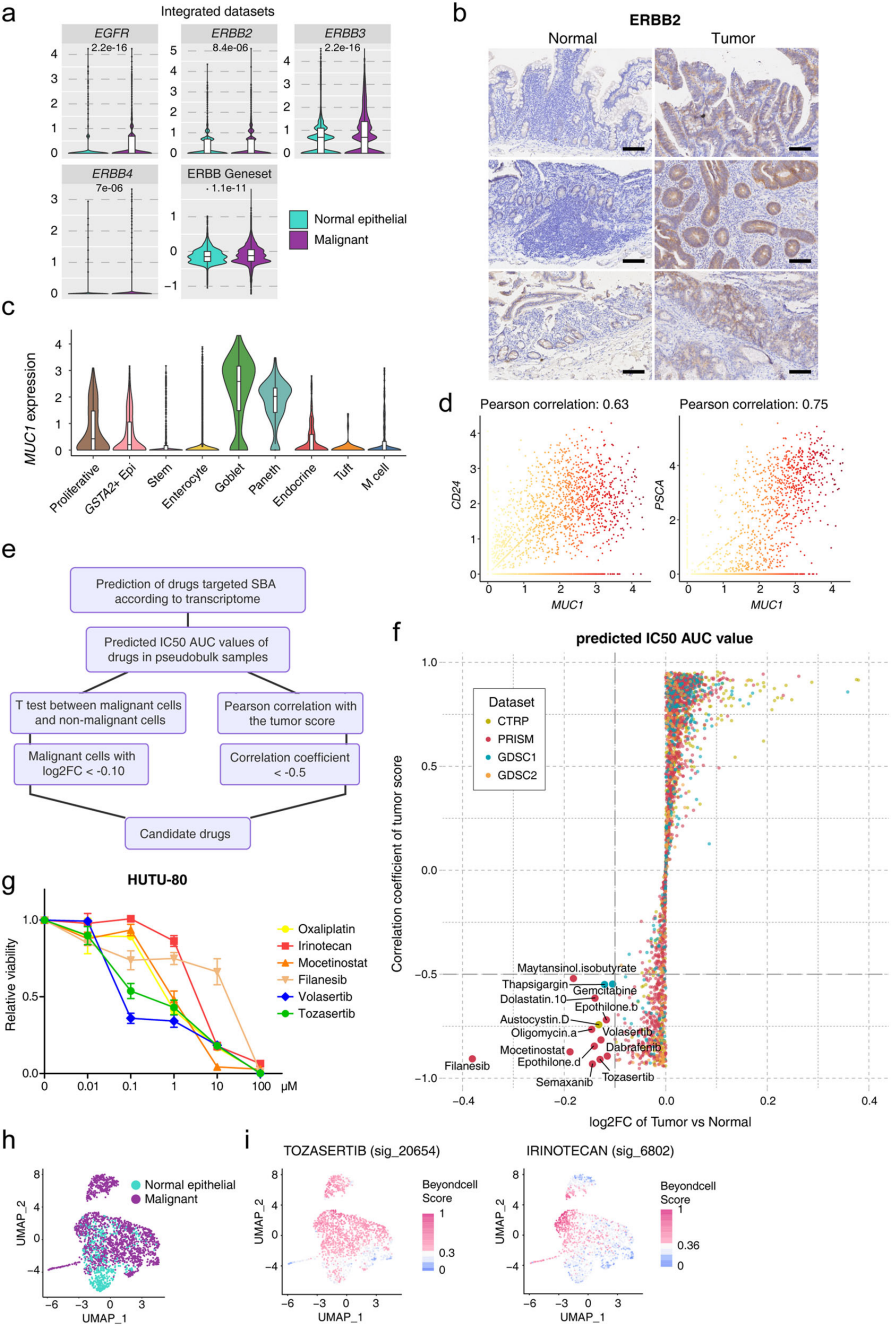
Identification of target genes and candidate drugs for SBA. **a** Violin plot exhibiting expression levels of key genes in the ERBB pathway and the ERBB family gene set score in the malignant cells and normal epithelial cells in the integrated dataset of the STRT and 10× datasets, with “ns” representing no significance and *P* values calculated by *t*-test. **b** Representative IHC staining of ERBB2 in adjacent normal and primary tumor tissues (original magnification 100×). The rows of the paired normal and tumor samples are from the same patients, and three individual patients are listed. Scale bar, 100 μm. **c** Violin plot showing *MUC1* expression in normal epithelial types from the 10× dataset. **d** Scatterplot exhibiting the positive correlation of expression of *MUC1* and other genes. **e** The brief workflow of drug predication. **f** Scatterplot exhibiting the log_2_FC of tumor sample compared with normal samples and correlation with tumor score according to the predicted IC_50_ AUC value of every drug. Drugs with labels were selected as candidate drugs. **g** Cell survival curve for HUTU-80 cells treated with the indicated inhibitors with a dose escalation from 0 to 100 μM. Data are presented as means ± SD. **h** UMAP plot identified the differences of malignant cells and normal epithelial cells based on transcriptional changes induced by drugs from databases built in Beyondcell. **i** Drug sensitivity of tozasertib and irinotecan evaluated in single cells from the STRT dataset based on Beyondcell.

The available types of treatments for SBA are limited and there is a lack of specific clinical therapies for SBA. Previous studies showed frequent somatic mutations of SBA, such as *ERBB2*, *KRAS*, *PTEN*, *PIK3CA*, *IDH1*, which may be potential targets for SBA treatments. Although we did not detect most of somatic mutations in the above genes according to the WES analysis, we sought to evaluate the expression levels of these potential targets. We identified the ERBB family genes, including *EGFR*, *ERBB2*, *ERBB3* and *ERBB4*, that were specifically upregulated in malignant cells in these two datasets (Fig. 7a; Supplementary Fig. S11a). Previous studies have reported that *ERBB2* is frequently mutated in SBA, which is associated with worse clinical outcomes^7,8^. The IHC staining of *ERBB2* showed the higher protein expression levels in tumor tissues than normal tissues in SBA (Fig. 7b). As for other frequently mutated genes, though the absolute expression levels of these targets are relatively low, the expression levels of *KRAS*, *PIK3CA*, *PTEN* are higher in malignant cells compared to normal epithelial cells, and the expression of *IDH1* in malignant cells and normal epithelial cells has no significant differences (Supplementary Fig. S11b).

Among the DEGs identified in malignant cells in SBA, *MUC1* is widely expressed in different patients, and the IHC staining confirmed the specificity of MUC1 in tumor tissues of SBA (Fig. 2d), which indicates that *MUC1* can be regarded as a universal marker of SBA according to our scRNA-seq data. *MUC1* is a member of the mucin family and was reported to be highly expressed in gastrointestinal cancers^31^, which was previously found to be associated with EMT in SBA. *MUC1* is also associated with oncogenes such as *EGFR*, *ERBB2* and T cell exhaustion-related genes such as *LGALS3* functionally and physically according to the protein interaction network (Supplementary Fig. S11c). As for normal intestinal epithelial cells in both the STRT and 10× datasets, *MUC1* was expressed mainly in goblet cells (Fig. 7c; Supplementary Fig. S11d, e). The expression of *MUC1* was positively correlated with cancer stem cell markers such as *CD24*, *PSCA* and *TM4SF1*, which indicated that the high expression of *MUC1* in malignant cells was associated with tumor proliferation (Fig. 7d; Supplementary Fig. S11f). To explore more potential functions of *MUC1* in gastrointestinal cancers, the bulk RNA-seq data and protein expression data using the reverse phase protein array (RPPA) method of CRC from the TCGA dataset were used, and we identified the correlation of expressed genes at RNA and protein levels with the *MUC1* RNA expression (Supplementary Fig. S11g). The common highly-correlated genes with *MUC1* RNA expression both at RNA and protein levels, such as *PARP1*, *MTOR*, *MAP3K6*, were found, which gives clues for the molecular mechanisms of *MUC1* for tumorigenesis of SBA. Furthermore, *MUC1* was reported as an attractive candidate target in many CAR-T studies. Although CAR-T therapies were mainly provided to be effective in blood cancers rather than solid tumors, the CAR-T therapy of *MUC1* for SBA may be developed and further studied to benefit SBA patients.

### Prediction and verification of specific drug candidates for SBA based on transcriptomic analyses

To identify more specific drug candidates for SBA, we used a ridge regression-based method to predict the potential drug responses of SBA^32,33^ (Fig. 7e). Drug sensitivity data in the form of IC_50_ AUC values and gene expression profiling data of CCLE cell lines were used as training sets, including data on 2521 drugs and 1969 cell lines^34–38^ (Supplementary Fig. S12a). We utilized the log_2_FC values of tumor cells relative to normal cells and the correlation coefficients of tumor scores as the filtering criteria to predict potential candidate drugs for SBA. Fourteen predicted drugs were obtained, including one from the CTRP database and two from the GDSC1 database, while the others were from the PRISM database (Fig. 7f; Supplementary Table S7). Of the fourteen predicted drugs, we selected four of them including filanesib, mocetinostat, tozasertib and volasertib to verify the inhibitory effects for SBA. Filanesib is a kinesin spindle protein (KIF11) inhibitor which has recently been proposed for cancer treatment, specifically for multiple myeloma. Mocetinostat is a histone deacetylase (HDAC) inhibitor undergoing clinical trials for treatment of many cancers including follicular lymphoma, Hodgkin’s lymphoma and acute myelogenous leukemia. Tozasertib is a pan-Aurora inhibitor, mostly against Aurora A. Volasertib is an experimental small-molecule inhibitor of the PLK1 (polo-like kinase 1) protein and is developed for use as an anti-cancer agent. The criteria of selecting these candidates are as follows. Firstly, these selected drug candidates were at least in the phase II of clinical traits in other cancer types, which means that the toxicity and side effects are acceptable for cancer patients and the safety- and dose-related research data are available. Secondly, these drugs are relatively easy to obtain. Although the absolute expression levels of these genes are relatively low, we can see targets of these four selected drugs are more highly expressed in tumor tissues compared to adjacent normal tissues (Supplementary Fig. S12b).

To assess the inhibitory effects of candidate drugs for SBA, we screened the HUTU-80 cell line, which was derived from duodenal adenocarcinomas, with these four drugs including filanesib, mocetinostat, tozasertib and volasertib as well as two conventional drugs of SBA, oxaliplatin and irinotecan as controls (Fig. 7g; Supplementary Table S8). Especially at the concentration of 0.1 μM, the inhibitory effects of volasertib and tozasertib were significantly higher than oxaliplatin and irinotecan (Supplementary Fig. S12c). Although moncetinostat also had a comparable inhibitory effect at the concentration of 0.1 μM, it did not show stronger effects at higher concentrations. We performed the cell viability assay and the dose-response analysis of these drugs to detect inhibitory effects. In HUTU-80 cells, volasertib and tozasertib had the most pronounced inhibitory effects, whereas moncetinostat and silanesib did not show prominent differences of inhibitory effects compared to oxaliplatin and irinotecan. Both volasertib and tozasertib target cell cycle to inhibit cell division^39,40^. To clarify susceptibility of screened drugs in separate malignant cells of SBA, we calculated drug responses of tozasertib and irinotecan of separate epithelial cells in our STRT dataset. The UMAP embeddings were computed by transcriptional changes induced by drugs from databases built in Beyondcell^41^, and clusters were presented according to malignant cells and normal epithelial cells, which is similar to clusters based on gene expression (Fig. 7h). Tozasertib has three similar related signatures in the built-in datasets of Beyondcell. High and low drug susceptible scores of these signatures of tozasertib were similar to the classification of malignant and normal epithelial cells, which was concordant with our prediction results and proved the drug specificity in malignant cells of SBA (Fig. 7i; Supplementary Fig. S12d). Irinotecan only showed the high susceptibility in part of malignant cells, and tozasertib was highly susceptible in almost all the malignant cells, which may explain the higher inhibitory effect of tozasertib compared to irinotecan (Fig. 7i). Irinotecan is one of the conventional drugs for intestinal cancers^42^, and the heterogeneity of drug susceptibility of irinotecan in SBA indicated that more specific drugs are in urgent need for SBA. The high susceptibility and specificity of tozasertib may provide new clues for treatments of SBA.

## Discussion

The knowledge and available data about SBA are limited, and extrapolations from studies of CRC are used as the major reference in the treatment of SBA^4,5,43^. In our study, several important conclusions are presented. In SBA, we identified 4 prevalent subtypes of malignant cells: the cell cycle program, the mitochondria program, the metabolism program and the EMT program. The progression trajectory of these 4 subtypes of malignant cells is from the cell cycle program to the mitochondria program, and progressing into either the metabolism program or the EMT program. These 4 prevalent subtypes of malignant cells and their progression trajectory are identified in two datasets, which represents the generalization of the finding. This finding represents the intra-tumor heterogeneity of SBA malignant cells and the scRNA-seq strategy is ideal for analyzing intra-tumor heterogeneities. As for tumor immune microenvironment, we identified that the EMT-program malignant cells have a high association with highly exhausted CD8^+^ T cells. The interactions between EMT-program malignant cells and highly exhausted T cells may reveal the association between the EMT signature of malignant cells and T cell exhaustion in SBA. With published scRNA-seq data of other gastrointestinal cancers, we compared gastric, duodenal, jejunal and colorectal malignant cells at the global gene expression levels, and revealed the closer relationship between GC and duodenal subtype of SBA and closer relationship between jejunal subtype of SBA and CRC. These ordered relationships are also in accordance with the spatial order of the gastrointestinal tract. Because the current therapies of SBA mainly refer to CRC, it raises an issue whether it is an optimal way to treat duodenal cancers with therapies of CRC. Based on the single-cell transcriptome signatures of malignant cells and normal epithelial cells, published drug treatment resources and in-depth bioinformatics analyses, specific candidate drugs of SBA were predicted. Verifications of predicted drugs were performed in the HUTU-80 duodenal cancer cell line, and inhibitory effects of volasertib and tozasertib are stronger than classical clinical drugs for SBA such as oxaliplatin and irinotecan when used at the same concentration. These predicated and verified drug candidates may potentially benefit SBA patients based on the current situation that there lacks specific drugs for SBA.

The case number is only 12 due to the rarity of SBA, which is one of the limitations of this study. To maximize the utility of this study, we collected 34 samples from these 12 SBA patients, with 21 primary tumor samples, 10 adjacent normal samples and 3 lymph node metastatic tumor samples (Supplementary Table S1). Previous studies about SBA mainly focused on genomic mutation information, which can be captured from frozen tissues or paraffin sections. High-quality single-cell transcriptome data can only be obtained from fresh tissues, and the rarity of SBA limits the number of collected samples to make a large cohort of single-cell transcriptome information of SBA. Although the number of patients of our dataset is limited, the numbers of the tumor and adjacent normal tissue samples are acceptable to reveal intra-patient heterogeneities of SBA. As for the number of ileal samples in this study, the low number of ileal patients is due to much lower percentage of ileal cancers in SBA. And it is more difficult to collect a relatively large number of ileal cancer samples. Although the number of patients of ileal cancer is only 2, there are 4 primary tumor samples and 2 adjacent normal samples, which is acceptable to identify intra-patient tumor heterogeneities. To compensate for the limitation, we included published single-cell transcriptome data from two normal ileum samples to increase normal ileal cell numbers and make related biological conclusions more representative. Meanwhile, more studies and more samples for SBA were needed, and we think that more samples will bring more findings and verify our findings in the future.

In this study, we used two different library construction methods, the modified STRT method and 10× Genomics method to balance the number of captured genes in each individual cell and cell throughput for each run. The modified STRT method can capture more genes with a median number of 4187 detected genes in each cell, which is much higher than the number of captured genes of the 10× Genomics method with a median number of 1710 detected genes in each cell (Supplementary Fig. S1a). The 10× Genomics method is the mainstream high-throughput scRNA-seq method, and the number of captured cells of the 10× dataset is 33,374 while the number of captured cells of the STRT dataset is 3676. Combining these two scRNA-seq methods, the number of detected genes and the number of captured cells were well balanced to explore more information in individual cells and enough number of cells to determine cell types. The logic of our analysis is using these two datasets as individual datasets to find the biological patterns first in one dataset and then confirm them in the other dataset. We also use distinct advantages of these two datasets to draw dataset-specific conclusions. As for specific conclusions from the STRT dataset, we used the STRT dataset to predict potential specific drugs for SBA because the STRT dataset capture more genes than the 10× dataset and can reduce the influences of dropout effects. As for specific conclusions from the 10× dataset, we only obtained enough number of T cells in the 10× dataset and explored T cell signatures using the 10× dataset. We also used the 10× dataset to identify the relationship of gastrointestinal caners because there are less batch effects between this dataset and the published GC and CRC 10× datasets.

We first revealed the molecular landscape of SBA at the single-cell level. We used two methods to capture single-cell transcriptome of SBA, and the integration of normal epithelial cells proved the consistency of these two datasets. We also combined bulk transcriptome data and performed the deconvolution analysis to infer increasing percentages of fibroblasts and decreasing percentages of epithelial cells in tumor tissues, which indicates the potentially important role of fibroblasts in SBA. We identified biomarkers of SBA and potential target genes for its treatment, such as *CEACAM5* and *MUC1*. *CEACAM5* is a traditional marker used in the clinical diagnosis of many types of cancers including SBA^44^, and *MUC1* is a tumor antigen that is also targeted in many clinical traits of immunotherapies^31,45^. *MUC1* presents a positive correlation with stem-related markers in SBA and is also reported to show good druggability^46,47^. Furthermore, almost all the physiological functions of small intestine were observed to be decreased in the malignant cells, as expected, and different metabolic pathways were enriched in malignant cells and normal epithelial cells of different intestinal regions, which may be meaningful for related clinical treatments.

To further investigate the intratumoral heterogeneity and subtypes of SBA, four common gene expression programs of the malignant cells were identified in the patients. Malignant cells with a strong cell cycle program first progress into an intermediate state with mitochondria-enriched programs, and then to two cell fates, the metabolism-enriched program and the EMT-enriched program, emerging as the terminal progression states of the malignant cells. The EMT program was speculated to be an endpoint of tumor progression without the expression of classical EMT markers. The progression trajectory started from the cell cycle program, through the mitochondria program and progressing into either the metabolism program or the EMT program. Meanwhile, the STRT and 10× datasets both represent these 4 subtypes of malignant cells and their progression relationship, which manifests the generalization of these two datasets. We inferred that the specific EMT signature of SBA represented the initial stage of EMT based on the GRN analysis. The reason for the lack of classical EMT markers in the identified EMT program of SBA is unclear but may have resulted from the diversity of tumor progression and specific mechanisms. Markers of this process were also shared in non-classical EMT programs of other cancer types, indicating the similar signatures among diverse types of cancers^17^.

Furthermore, we identified the connections of malignant cells with fibroblasts and T cells in the tumor microenvironment. As for fibroblasts, several studies hinted that fibroblasts are important in gastrointestinal cancers, and we want to capture enough fibroblasts to explore related phenomena. Although we tried to enrich fibroblasts in P5 and P6, the ratio of fibroblasts from the enriched sample (97/1237) is not significantly different from the ratio of fibroblasts in unenriched samples (242/2439). Although the attempt of enriching fibroblasts is unsuccessful, the quality of the cells (such as gene and transcriptional molecule numbers) is not influenced and the cells can still be used for downstream analyses. There were four common fibroblast subclusters, cluster C1, cluster C2, proliferative fibroblasts and myofibroblasts in both the STRT and 10× datasets, which represents the universality and representativeness of the identified fibroblast subclusters. Only the common fibroblast subclusters were analyzed further in our study to make our conclusions more reliable. The identified fibroblast subcluster C1 was also reported in other scRNA-seq studies, such as a single-cell inflammatory bowel disease study and a single-cell colorectal cancer study^30,48^. Furthermore, we identified continuously changing interactions in fibroblasts during tumor progression. As we found and confirmed increased percentages of fibroblasts in tumor tissues of SBA, and these interactions may be involved in tumor progression and metastasis. For T cells, we focused on T cell exhaustion, which was revealed in both CD4^+^ and CD8^+^ T cells from tumor tissues. Among CD8^+^ T cells, highly exhausted T cells tended to interact more with EMT-program malignant cells, involving interaction pairs such as *HMGB1–HAVCR2*, *CEACAM1–HAVCR2*, *HBEGF–CD44* and *LGALS3–LAG3*. The similar phenomena were also reported previously and supported by studies of other cancer types^49–51^. More verifications and studies are required for these phenomena in the future. The interactions of ex-high T cells and EMT-high malignant cells were not significantly enriched in GC or CRC, which indicated that this EMT-program malignant cell*–*highly exhausted CD8^+^ T cell interaction signature may be specific to SBA.

Based on the current situation of limited treatments for SBA, we utilized our data and published data to evaluate and screen for potential drugs. We compared GC and CRC with SBA and found that duodenal subtypes and GC were more closely related at the transcriptomic level. It should be noted that treatments for CRC are widely referenced for the treatment of SBA^5^; therefore it should be further considered whether these therapies are suitable for duodenal cancers. Although a previous study showed a closer relationship of SBA with CRC than with GC at the CNV level, we think that different dimensions of data may have different features, and it is reasonable about the inconsistency between the CNV-related features and the gene expression-related features. In that study, SBA was similar to CRC at the CNV level, but the differences of individual patients may result from the different genomic mutations. Furthermore, previous SBA studies were based on bulk sequencing, in which signatures of malignant cells from SBA may be covered up by tumor microenvironment cells. Combing with our finding that proportions of fibroblasts increase significantly in tumor samples of SBA, we think that microenvironment cells of tumor samples influence the evaluation of CNV to some extent. As for our single-cell transcriptome dataset, we separated epithelial cells from all the microenvironment cells of GC, SBA and CRC, and selected out malignant cells from normal epithelial cells to represent more specific signatures of different cancer types. In addition, we also divided SBA into duodenal, jejunal and ileal malignant cells, which makes the comparison more accurate. As the spatial order of the gastrointestinal tract from stomach, duodenum, jejunum to colorectum, we found that duodenal malignant cells were more similar to gastric malignant cells based on HVGs, which is corresponding to the spatial distance. As for SBA and CRC, combining with signaling pathway analysis, the EMT signatures of SBA were found to be weaker than those of CRC at the global transcriptome level, which may explain the initial EMT signature identified in SBA and a more mature EMT signature in CRC. Based on the identified complex molecular subtypes of CRC and potential molecular subtypes of SBA, more studies are needed to reveal the EMT signature in gastrointestinal cancers in the future.

Moreover, gene expression profiling data and drug response IC_50_ values were integrated to predict potential drug candidates of SBA. There are relatively few SBA patients, and thus large-scale preclinical or clinical trials are lacking. By using published omics and drug response data, we screened out 14 potential drugs for SBA treatment. The cell viability experiment confirmed that volasertib and tozasertib exhibit more powerful inhibitory effects for the duodenal cancer cell line than conventional drugs for SBA. The high sensitivity and specificity of tozasertib for SBA were also identified in our analysis. Volasertib and tozasertib were predicted through combing our scRNA-seq data and published data and verified to be effective in a duodenal cancer cell line, which may benefit SBA patients. More studies and clinical data for these drugs are worth performing in the future.

We performed a large set of verification experiments to confirm our major conclusions. First, the cell viability assay in the HUTU-80 cell line for the predicted drugs were performed. Inhibitory effects of volasertib and tozasertib are stronger than those of classical clinical drugs for SBA such as oxaliplatin and irinotecan when used at the same concentration, which indicates that the prediction of drug candidates is reliable and effective. Second, we used the IHC staining to verify many markers identified in the study. As for malignant cells, we found several DEGs or markers in malignant cells. MUC1 as a DEG of malignant cells, proved to be expressed more highly in malignant cells than normal epithelial cells by the IHC staining. ERBB2 is found to be highly expressed in malignant cells in SBA, which is also reported to be highly expressed in SBA, and the IHC staining of ERBB2 also confirmed our conclusion. As for fibroblasts, we used the deconvolution analysis to reveal the higher proportion of fibroblasts in tumor tissues compared to adjacent normal tissues, and we used the marker of fibroblast, TAGLN, to verify the higher proportion of fibroblasts in tumor tissues compared to normal tissues. We also found that the ratio of myofibroblasts increases in tumor during SBA tumorigenesis, and the IHC staining of ACTA2, which is a well-known marker of myofibroblasts, confirmed the higher proportion of myofibroblasts in SBA tumor tissues. The DEGs of fibroblasts from tumor tissues compared to adjacent normal tissues were also identified, and one of them, RCN3 is also verified by the IHC staining. As for T cells, the exhaustion signature was found in CD8^+^ T cells. The exhaustion marker, TIM3 was highly expressed in T cells of tumor tissues compared to adjacent normal tissues, which is also confirmed by the IHC staining. However, more functional experiments for SBA are needed in the future.

In summary, our work systematically studied the characteristics of malignant cells and tumor microenvironment cells in SBA, revealing signatures of pathological phenotypes, the connections between malignant cell and microenvironment cell signatures and drug candidates with related targets. SBA is rare but has high mortality rate, and large-scale clinical trials of SBA are difficult to carry out. The enhanced cell–cell interactions between EMT-program malignant cells and highly exhausted T cells may provide clues regarding immunotherapies for SBA. The prediction and verification of approved drugs that may be used to treat SBA could guide clinical treatment decisions.

## Materials and methods

### Human specimen sampling

The research was approved by the Ethics Committee of Peking University Third Hospital (License# IRB00006761-M2016170). Informed consents of this study were signed by all the patients. Small bowel cancer tissues were sampled from 12 small bowel cancer patients, who received small bowel resection in Department of General Surgery, Peking University Third Hospital. Normal mucosae at least 5 cm from tumor border were sampled, and lymph node metastases from 3 patients were also sampled. Sampling was performed immediately after surgical resection to retain cell activity.

### Single cell isolation

Both normal mucosae and cancer tissues were cut into pieces and digested with the mix of collagenases (2 mg/mL collagenase II and IV; Invitrogen), followed by incubation on a shaker at 37 °C until no visible pieces were found in the digestion solution. CD90 antibody (BioLegend, 328110) was used in FACS to screen CD90^+^ fibroblasts.

### Single-cell cDNA amplification and library construction

The modified STRT method was applied to perform cDNA amplification and library construction, which has been described in detail in previous studies^11,12^. The library was prepared using a KAPA Hyper Prep Kit (KAPA Biosystems) and sequenced using the 150-bp paired-end sequencing method on Illumina HiSeq 4000 platforms. As for the 10× method, single cell separation, DNA amplification and library construction were performed following the manufacturer’s guidelines of chromium single-cell sequencing technology from 10× Genomics. The scRNA-seq libraries were constructed using the Chromium Single Cell 3ʹ Library and Gel Bead Kit V3. Finally, the prepared libraries were sequenced on Illumina NovaSeq 6000 platforms.

### WES

About 200 ng genomic DNA was extracted and fragmented by sonication to 150–200 bp length. Then end repair and ligation by adaptors were performed, fragmented DNA with adapters were subjected to PCR amplification with the mix of NEB universal primer, NEB index primers and 2× KAPA HiFi HotStart ReadyMix (Kapa Biosystems, Cat# KK8054). Finally, cDNA libraries were prepared to be captured by WES using SureSelectXT Human All Exon v6 kits (Agilent Technologies, Cat# G7530-9000).

### IHC staining

Normal mucosae and cancer tissues were fixed in 10% neutral buffered formalin for 24 h, and then embedded in paraffin. Paraffin-embedded tissues were sectioned into 5 μm-thick slices. The slides were incubated with primary antibody including anti-TAGLN antibody (abcam, ab155272, 1:800), anti-ACTA2 antibody (abcam, ab7817, 1:2000), anti-RCN3 antibody (atlas antibodies, HPA043134, 1:500), anti-ERBB2 antibody (ZSGB-Bio, ZA-0023), anti-MUC1 antibody (abcam, ab70475, 1:100) and anti-TIM3 antibody (abcam, ab241332, 1:1000) at 4 °C overnight and then incubated with secondary antibody (horseradish peroxidase-conjugated IgG, ZSGB-Bio PV-6000) at 37 °C for 30 min and visualized using diaminobenzidine (DAB).

### Cell culture

Human duodenum adenocarcinoma cell line HUTU-80 was obtained from Center of Basic Medical Research, Peking University Third Hospital. The cell line was routinely grown in Minimum Essential Medium (MEM) containing non-essential amino acids (NEAA) (41500, Solarbio, China) supplemented with 10% fetal bovine serum (10099-141, Gibco, USA) and penicillin (100 IU/mL)-streptomycin (100 mg/mL) solution (SV30010, HyClone, USA) and maintained at 37 °C and 5% CO_2_.

### Cell viability assay

HUTU-80 cells (6 × 10^3^ per well) were seeded in 96-well plates. After attachment to the plates and culturing in complete growth medium for 24 h, the cells were treated with Oxaliplatin, Irinotecan, Tozasertib, Volasertib, Mocetinostat and Filanesib (HY-17371, HY-16562, HY-10161, HY-12137, HY-12164, HY-15187, MedChemExpress, China) at different final concentrations ranging from 100 nM to100 μM using dimethyl sulfoxide (DMSO, 0219605580, MP Biomedicals, USA) or H_2_O as a solvent control. The number of viable cells was determined by CellTiter-Glo Luminescent Cell Viability Assay (G7571, Promega, USA) following the kit protocol 36 h later and all experiments were performed in triplicate.

### Processing of scRNA-seq data

As for the STRT sequencing data, UMI-tools (verion 1.0.0) were applied to extract barcodes and unique molecular identifiers (UMIs), and seqtk (version 1.3) was used to filter the low-quality reads. Then STAR (version 2.7.1a) was used to align these clean reads to the human GRCh38 genome. We used featureCounts (version 1.6.4) to count read uniquely mapped to the genomes, and UMI-tools were applied to quantify the UMIs. In the STRT dataset, we retained cells with more than 1000 detected genes, 10,000 transcripts and lower than 20% of mitochondrial genes. As for the 10× dataset, we used Cell Ranger (version 3.1.0) with default arguments to process raw data, and the human GRCh38 was used as the reference genome. In the 10× dataset, we retained cells with more than 500 detected genes, 1000 transcripts and lower than 50% of mitochondrial genes. In both the STRT and 10× datasets, UMIs per cell were normalized and transformed to generate gene expression values by log transforming. The log-normalized values were used in the downstream analyses.

### Cell clustering analysis

We mainly used Seurat (version 3.2.2) to perform the downstream analyses^52^. Batch effects caused by experiment batches should be reduced, and we used the R packages RSCORE (version 0.1.0) and Harmony (version 1.0) to correct batch effects^53,54^. To obtain different cell types, we performed RSCORE to remove batch effects, with the parameters “max_step = 20” and the human protein–protein interaction network 3.5.173 version from BioGRID. To classify subclusters in separate cell types such as epithelial cells, fibroblasts and T/NK cells, Harmony was used to remove batch effects with the default parameters. Finally, individual cells were clustered through a graph-based clustering approach of Seurat.

### Integrating with published datasets

We used the sctransform function to normalize our datasets as well as published datasets including our STRT and 10× datasets, the normal ileum dataset and other gastrointestinal cancer datasets, and utilized the CCA method to integrate these datasets. The default parameters were used, and the expression values in assays of SCT were used to identify cell clusters and calculate DEGs.

### DEG analysis and gene enrichment analysis

DEGs of different cell types were identified through the FindAllMarkers function of Seurat using the wilcox test and the fold change with the value 2. The detailed parameter “test.use = “wilcox”, min.pct = 0.25, logfc.threshold = log(2)”. DEGs of malignant cells and normal epithelial cells in the whole datasets were calculated with the same parameters. Only genes with the adjusted *P* values (based on bonferroni correction) < 0.01 were retained as DEGs of malignant cells and normal epithelial cells. DEGs of malignant cells and normal epithelial cells of separate patients were retained with the fold change of 1.5 to involve as many markers as possible. Metascape (http://metascape.org/) was used to perform gene enrichment analysis.

### Analysis of the WES data

As for the CNV analysis, low-quality and adapter-contaminated reads were reduced and trimmed by Trimmomatic (version 0.39), and then clean reads were aligned to the human GRCh38 genome using BWA (version 0.7.17). CNVkit (version 0.9.6) was used to estimate CNV, which outputted normalized copy ratios of DNA segments.

As for somatic mutations, germline mutations were called using HaplotypeCaller built in GATK (Genome Analysis Toolkit, Version 4.0.12). Somatic mutations were called with paired peripheral blood samples as control. Somatic mutation calling tools including Mutect2 (built in GATK), MuSE (version 1.0), Varscan2 (version 2.4.3), SomaticSniper (version 1.0.5), Strelka2 (version 2.9.10) and LoFreq (version 2.1.3.1) were also used to call somatic SNVs, and the outputs were filtered and integrated by SomaticSeq (version 2.8.1) with the criterion that SNVs detected by more than four tools were retained. The default parameters of these tools were used. Only the “PASS” somatic mutations were used for downstream analysis.

### CNV inference by scRNA-seq data and malignant cell identification

The R package InferCNV (version 1.1.3) was used to process scRNA-seq data^55^. In both the STRT and 10× datasets, epithelial cell from adjacent normal tissues were applied as references, and CNVs of epithelial cells from cancer tissues were inferred according to expression pattern. With the inferCNV outputs, we used the mean of squares of deviation as the measurement and the 90th percentile of normal epithelial cells as the threshold value to divide epithelial cells into high CNV levels and low CNV levels. Combining with the cluster information, clusters in which epithelial cells with high CNV levels were dominant were regarded as malignant cell manually, and other clusters were regarded as normal epithelial cells.

### GRN analysis

We used the R package SCENIC (version 1.1.3) to evaluate TF activities^13^. The GENIE3 method was used to detect correlations, and the dataset of motifs located 20 kb around TSS from the cisTarget database was used.

### Gene set signature and signaling pathway enrichment analysis

To evaluate gene expression signatures in single cells, we used the AddModuleScore function of Seurat to score the expression level of the specific gene sets at the single-cell level. We used the Gene Set Variation Analysis (GSVA) enrichment scores to describe enrichment scores of the hallmark gene sets through the R package GSVA (version 1.32.0)^56^. The input was the mean expression of separate datasets. The “ssgsea” method of GSVA package was used. As for the GSEA analysis, the python package gseapy (version 0.9.17) was used to perform the GSEA analysis. The parameters of “permutation_type = ‘gene_set’, permutation_num = 1000, method = ‘signal_to_noise’” were used.

### Quantifying metabolism activity at the single-cell resolution

The R package scMetabolism (version 0.2.1) was used to evaluate metabolism activities at the single-cell resolution^57^. The count data were input, the “VISION” method and the KEGG pathways built in scMetabolism were used.

### Signal entropy rate analysis

We used the R package SCENT (version 1.0.2) to estimate the ‘differentiation’ potency of single cells^16^. Signal entropy rate of single cells was calculated by the default parameters.

### Deconvolution analysis

We employed CIBERSORTx (https://cibersort.stanford.edu/index.php) to perform the deconvolution analysis^14^. The signature matrix was derived from DEGs of different cell types in our 10× dataset, and the normalized expression matrix was from a published dataset of a microarray experiment of SBA (GSE61465).

### Pseudotime analysis

We used the R package Monocle2 (version 2.12.0) with the DDR-Tree method and default parameters to perform the single-cell trajectory analysis^58^. The log-normalized data was used as the input. The DEGs or marker genes of clusters were applied as ordering gene sets. The cell trajectories were inferred after dimension reduction and cell ordering with the default parameters.

### Cell communication analysis

We used the python toolkit NATMI to perform cell communication analysis^22^. The log-normalized expression values and cell types corresponding to individual cells were used as the input data. The ExtractEdges function calculate expression and specificity of ligand–receptor interactions, and the DiffEdges function identifies changes of ligand–receptor interactions between two conditions. The built-in “lrc2p” database was used to predict interactions, the weight of edges was calculated by the mean method, and the detection threshold value is set to 0.2.

### Correlation with the expression of *MUC1*

The bulk RNA-seq data based on the HiSeq platform and protein expression data using the RPPA method of CRC from the TCGA dataset were used, and the analysis was performed by the LinkedOmics webtool^59^. As for the analysis for *MUC1*, the protein expression of MUC1 is not detected in the RPPA assay, and therefore we calculated the correlation of the RNA expression of *MUC1* and the other protein expression.

### Drug response prediction

We mainly used a ridge regression-based method of the R package pRRophetic (version 0.5) to predict the IC_50_ AUC values of potential drug responses^32^. The transcriptomic data of cell lines from CCLE and drug response data from CTRP, PRISM, GDSC1 and GDSC2 were utilized as training sets. Malignant and normal epithelial cells from epithelial cells of the STRT scRNA-seq data were divided into 50 groups randomly, and the mean expression values were used as the pseudo-bulk samples. We used the calcPhenotype function to calculate the IC_50_ AUC values for each corresponding drug, and selected drugs according to the following criteria: adjusted *P* values < 0.05 (under *t*-test), the log_2_FC value < −0.1, and the correlation with the tumor score < −0.5.

### Single-cell drug susceptibility assessment

The R package Beyondcell (version 1.2.1) was used to identify drug sensibilities of scRNA-seq data^41^. The drug perturbation signature collection (PSc) database built in Beyondcell was used. The number of detected genes per cell was corrected following the guidance.

### Analysis of the cell viability

GraphPad Prism software 5 was used to conduct the statistical analysis of cell viability. Data were presented as means ± SD.

## Supplementary information

Supplementary figures

Supplementary Table 1

Supplementary Table 2

Supplementary Table 3

Supplementary Table 4

Supplementary Table 5

Supplementary Table 6

Supplementary Table 7

Supplementary Table 8

## Data Availability

The raw sequence data reported in this paper have been deposited in the Genome Sequence Archive^60^ in National Genomics Data Center (National Genomics Data Center Members and Partners, 2020), Beijing Institute of Genomics (China National Center for Bioinformation), Chinese Academy of Sciences, under accession number HRA000483 that are publicly accessible at http://bigd.big.ac.cn/gsa-human.
