# Supplementary material for: Single-cell profiling reveals molecular basis of malignant phenotypes and tumor microenvironments in small bowel adenocarcinomas
Source: Cell Discov. 2022 Sep 14;8:92. doi: 10.1038/s41421-022-00434-x (PMC9475032; doi:10.1038/s41421-022-00434-x)
Supplement: Supplementary file 1 — Supplementary figures [file 41421_2022_434_MOESM1_ESM.pdf]

Supplementary Fig. S1

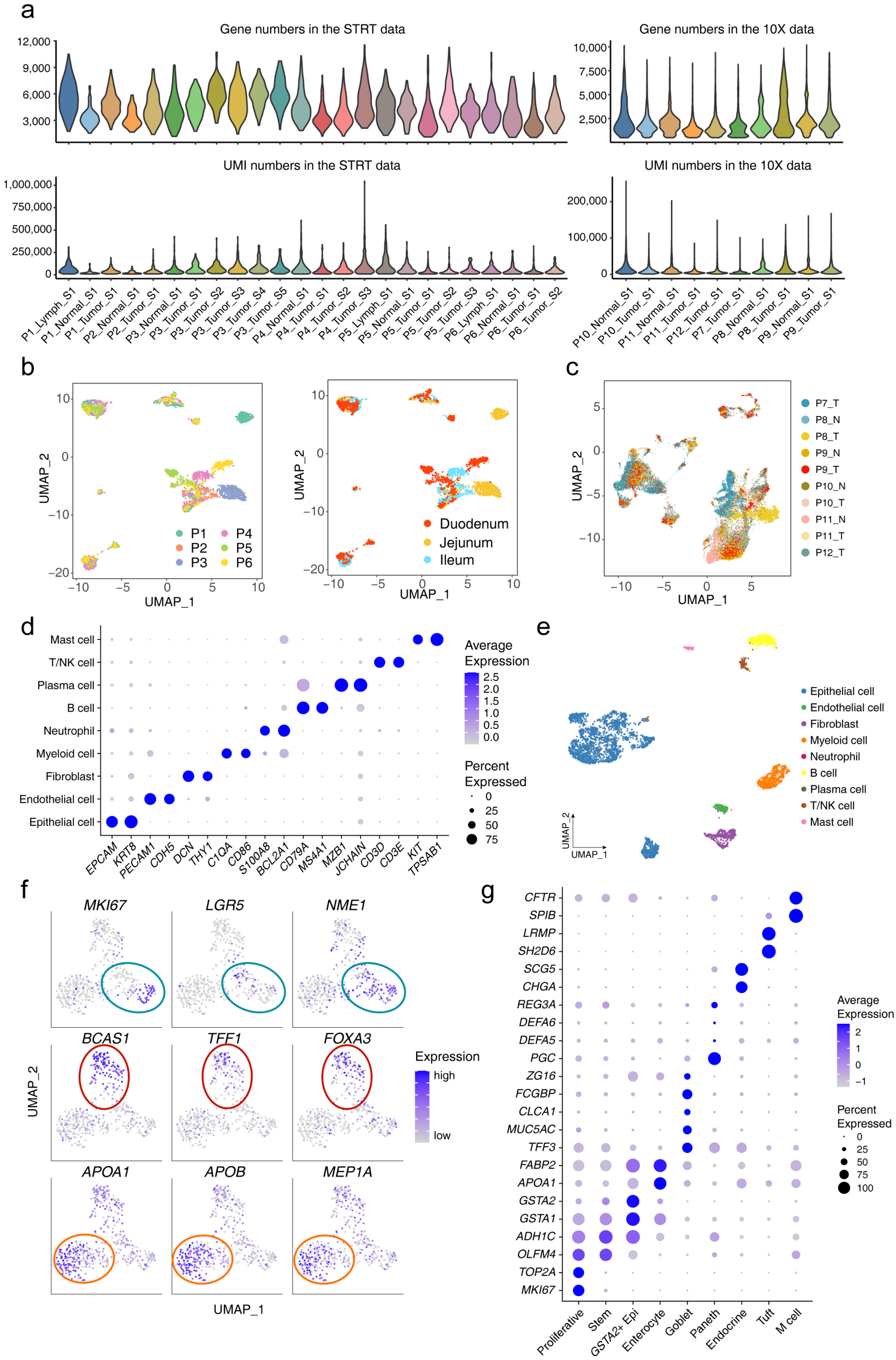

Supplementary Fig. S1 Global gene expression patterns of the STRT and 10× datasets in SBA. a Gene number and UMI number of the STRT and 10× datasets. **b, c** UMAP plot exhibiting the patient and intestinal region sources of the STRT and 10× datasets. **d** Dot plot presenting the normalized expression levels of corresponding markers of each cell cluster in the 10× dataset. **e** UMAP plot exhibiting the identified clusters based on TF scores of the STRT dataset. **f, g** UMAP feature plot and dot plot presenting the normalized expression levels of corresponding markers of normal epithelial cell clusters in the STRT and 10× datasets.

Supplementary Fig. S2

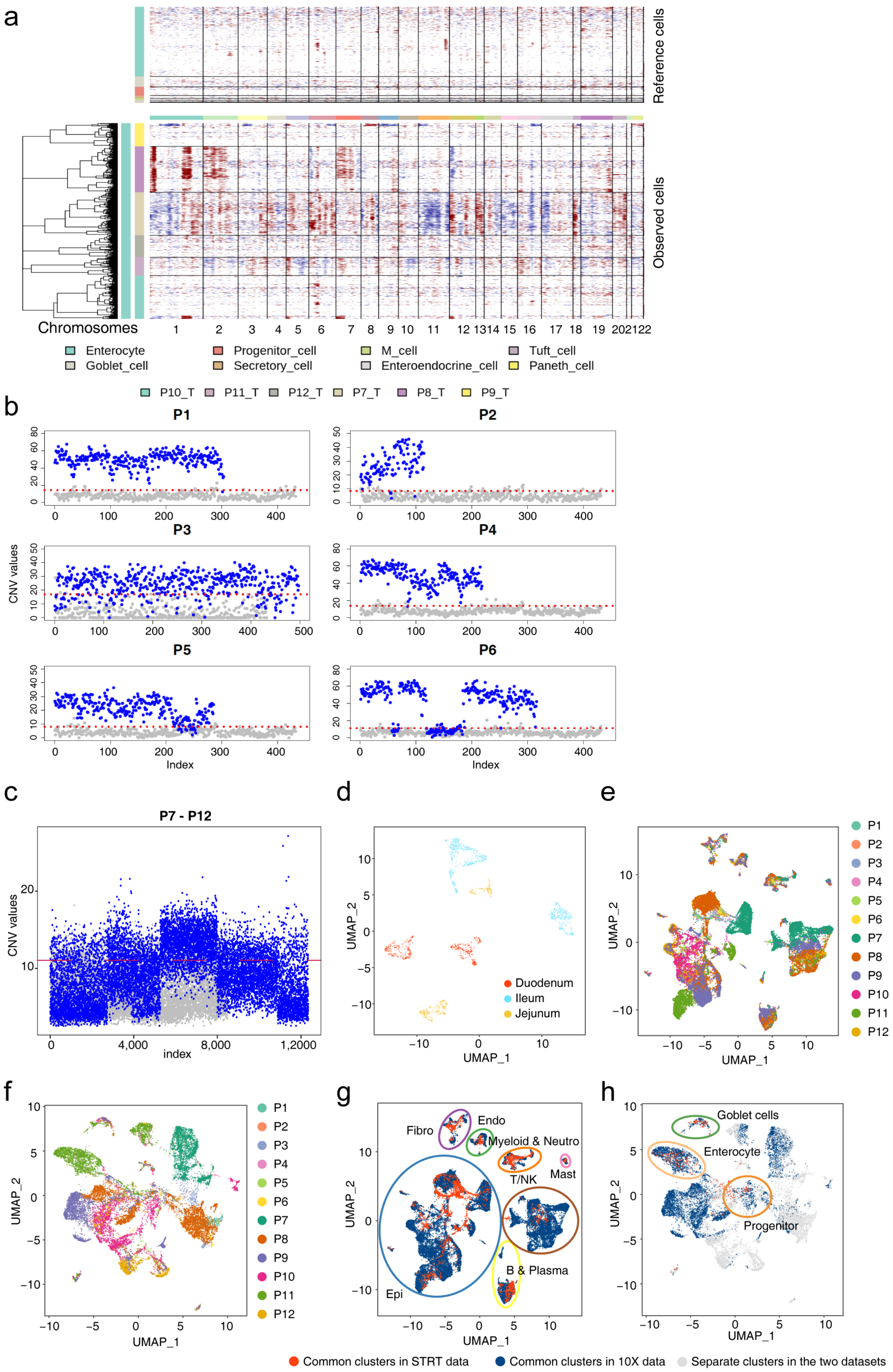

Supplementary Fig. S2 Inferred CNV features and integration of normal epithelial cells from the STRT and 10× datasets. a The upper panel exhibiting large-scale CNVs of single cells inferred based on normal epithelial cells in the 10× dataset. The lower panel exhibiting large-scale CNVs of single cells inferred based on epithelial cells from tumor tissues in the 10× dataset. **b**, **c** Scatterplot exhibiting the partition of the malignant cells with normal epithelial cells in the STRT and 10× datasets. **d** UMAP plot exhibiting cell clusters of the malignant cells from patients in the STRT dataset. **e**, **f** UMAP plot showing the integration of all the acquired cells and epithelial cells from the STRT and 10× datasets. **g**, **h** UMAP plot showing the integration of all the acquired cells and epithelial cells from the STRT and 10× datasets, with colors presenting the STRT or 10× datasets.

Supplementary Fig. S3

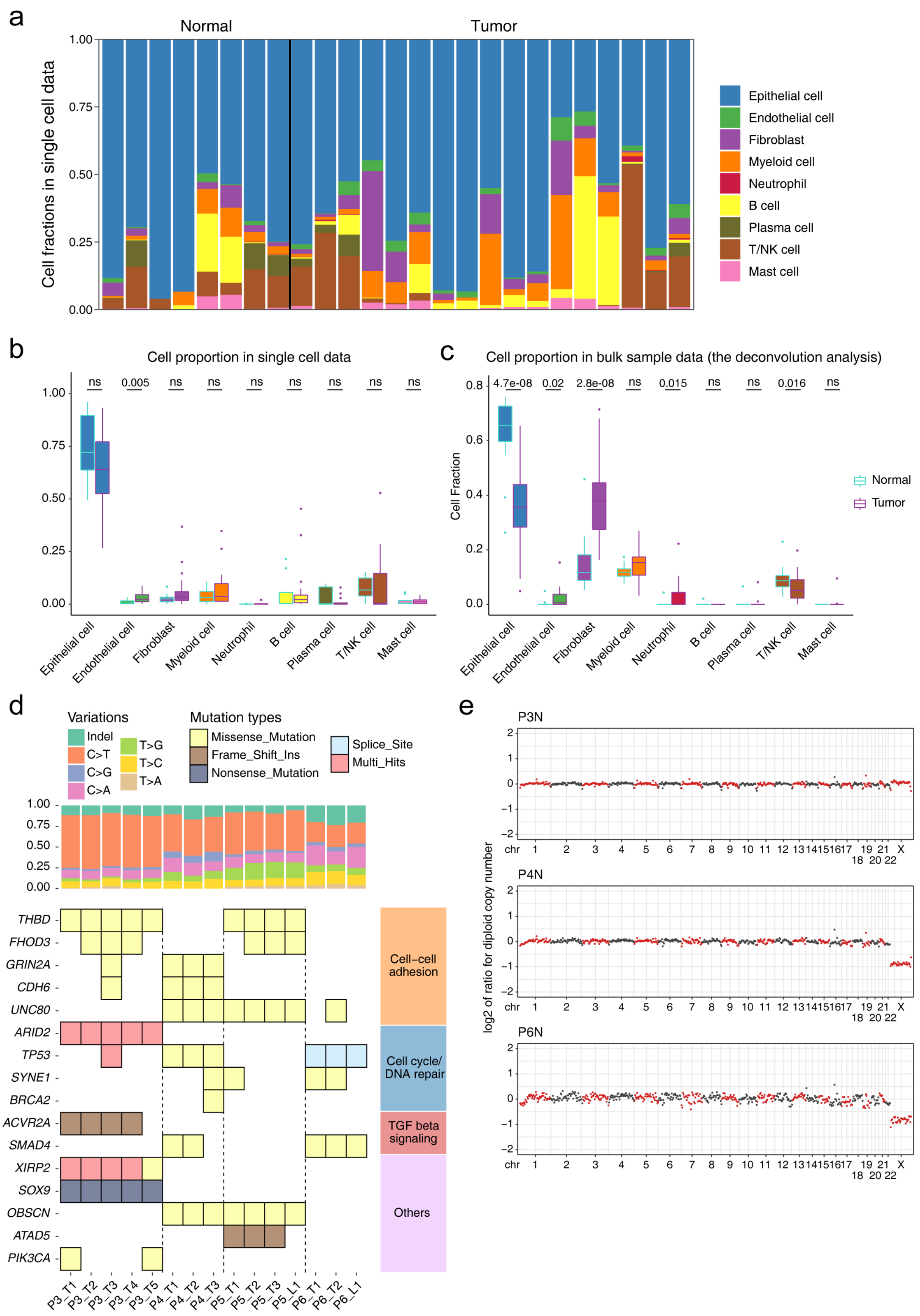

Supplementary Fig. S3 Proportions of different cell types and somatic mutations landscape of SBA. **a** Histogram showing cell type proportion of different cell clusters in the single-cell dataset. **b** Boxplot showing the cell composition percentage in the single-cell data, with "ns" representing no significance and *P* values calculated by *t*-test. **c** Boxplot showing the cell composition percentage inferred by the deconvolution analysis, with "ns" representing no significance and *P* values calculated by *t*-test. **d** The landscape of somatic mutations of samples from P3–P6. **e** The CNV plot representing the CNV values of normal epithelial samples from P3, P4 and P6.

Supplementary Fig. S4

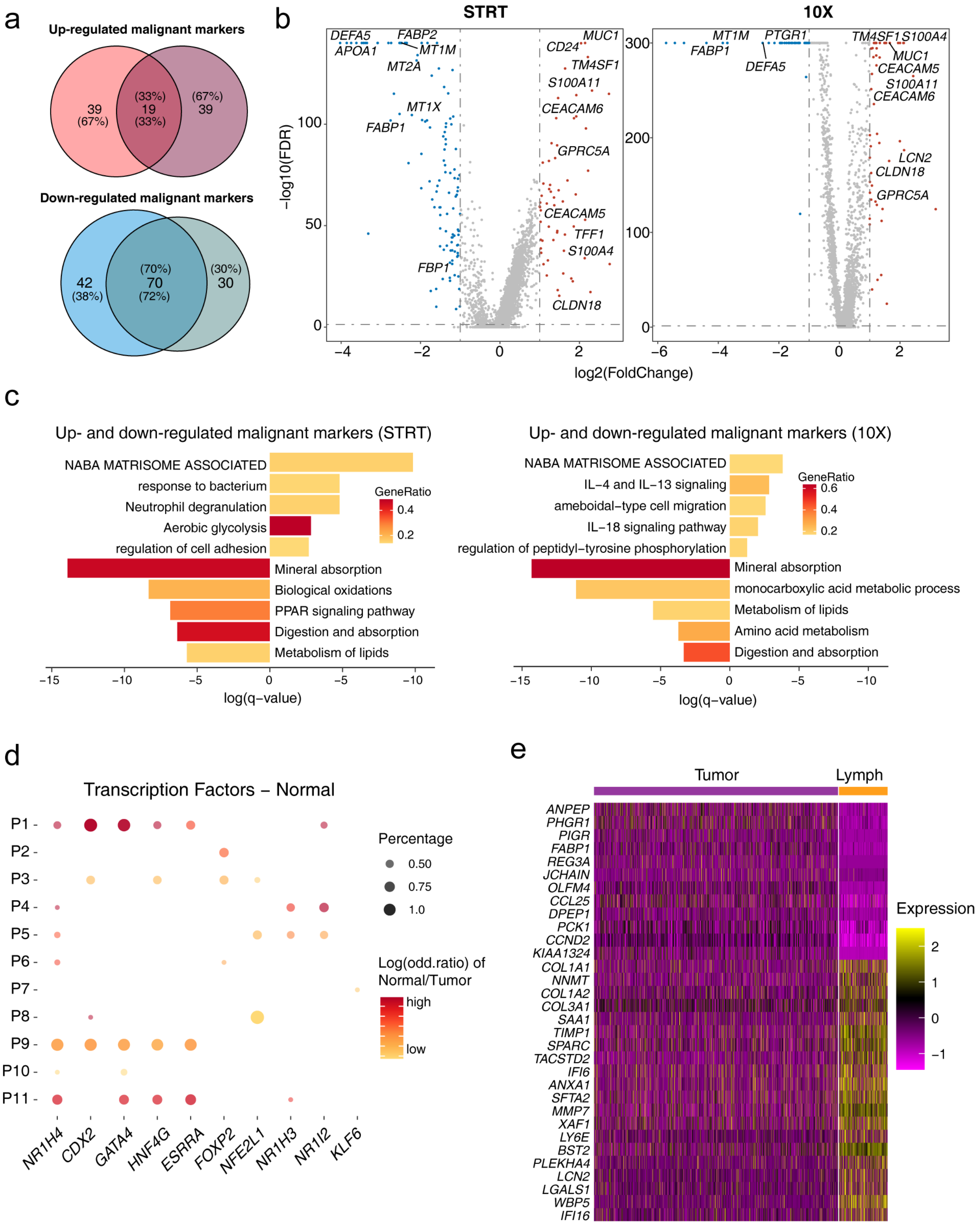

Supplementary Fig. S4 DEGs and Transcriptomic signatures of malignant cells of SBA. a Venn plot showing interactions of up- and downregulated malignant markers between STRT and 10× datasets. **b** Volcano plot exhibiting DEGs of the malignant cells compared with normal epithelial cells in STRT and 10× datasets. **c** Enrichment terms for up- and downregulated marker genes of the malignant cells in the STRT and 10× datasets. **d** Dot plot showing the log odd ratio values of normal/tumor of corresponding TFs, with *P* values calculated by χ^2 test. **e** Heatmap exhibiting DEGs of malignant cells from lymph node and primary tumor sites.

Supplementary Fig. S5

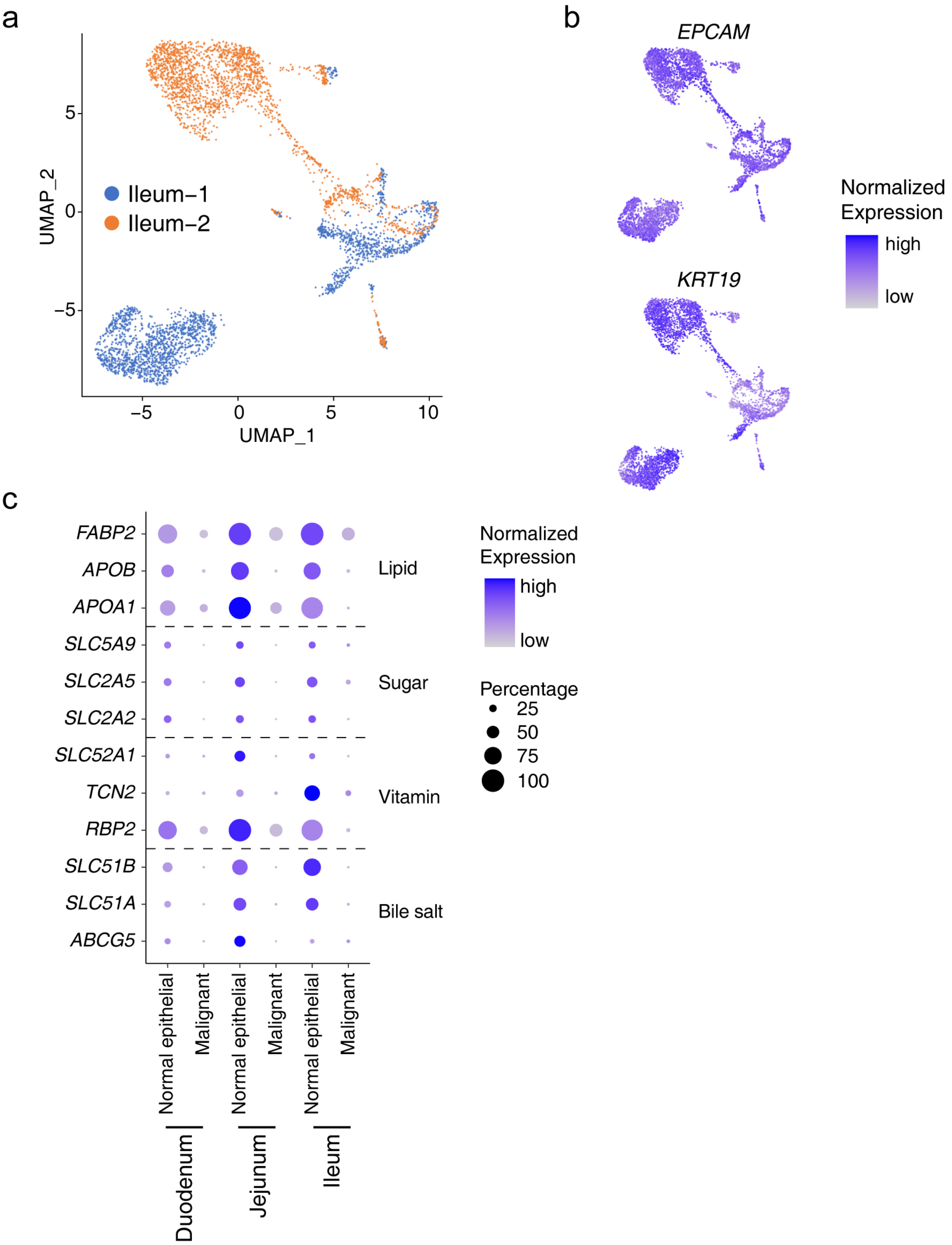

Supplementary Fig. S5 Analysis of the published ileal samples and the absorption and transportation related markers. **a**, **b** UMAP plot exhibiting the integrated datasets of two ileal normal samples with epithelial cell markers. The dataset is a subset of the GSE125970 dataset in the GEO database. **c** Dot plots presenting the normalized expression levels of absorption and transportation related markers in integrated datasets.

Supplementary Fig. S6

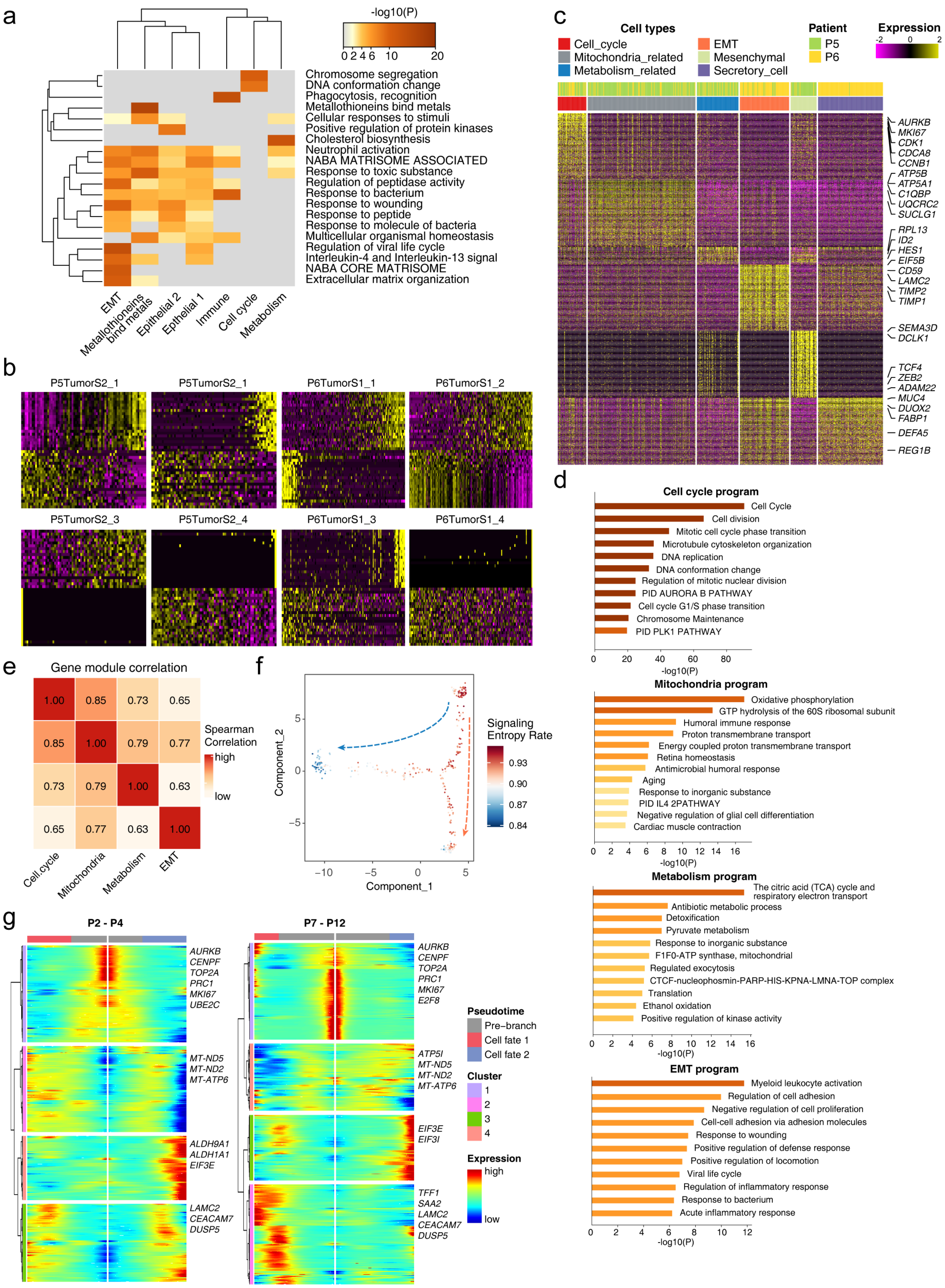

Supplementary Fig. S6 Validation of gene expression programs of malignant cells of SBA. a Heatmap showing enrichment terms for clustered expression signatures. **b** Heatmap exhibiting PC1–4 from the representative PCA results of P5_Tumor_S2 and P6_Tumor_S1 samples from P5 and P6. **c** Heatmap showing corresponding marker genes of the malignant cells related to gene expression programs in P5 and P6. **d** Enrichment terms for marker genes of identified gene expression programs. **e** Heatmap showing the Spearman correlation of the malignant cells belong to different expression programs. **f** Scatterplot showing the developmental trajectory of malignant cell labeled with the signaling entropy rate. **g** Heatmap showing the identified gene expression programs of the malignant cells along the pseudotime trajectory of P2–P4 in the STRT dataset and P7–P12 in the 10× dataset.

Supplementary Fig. S7

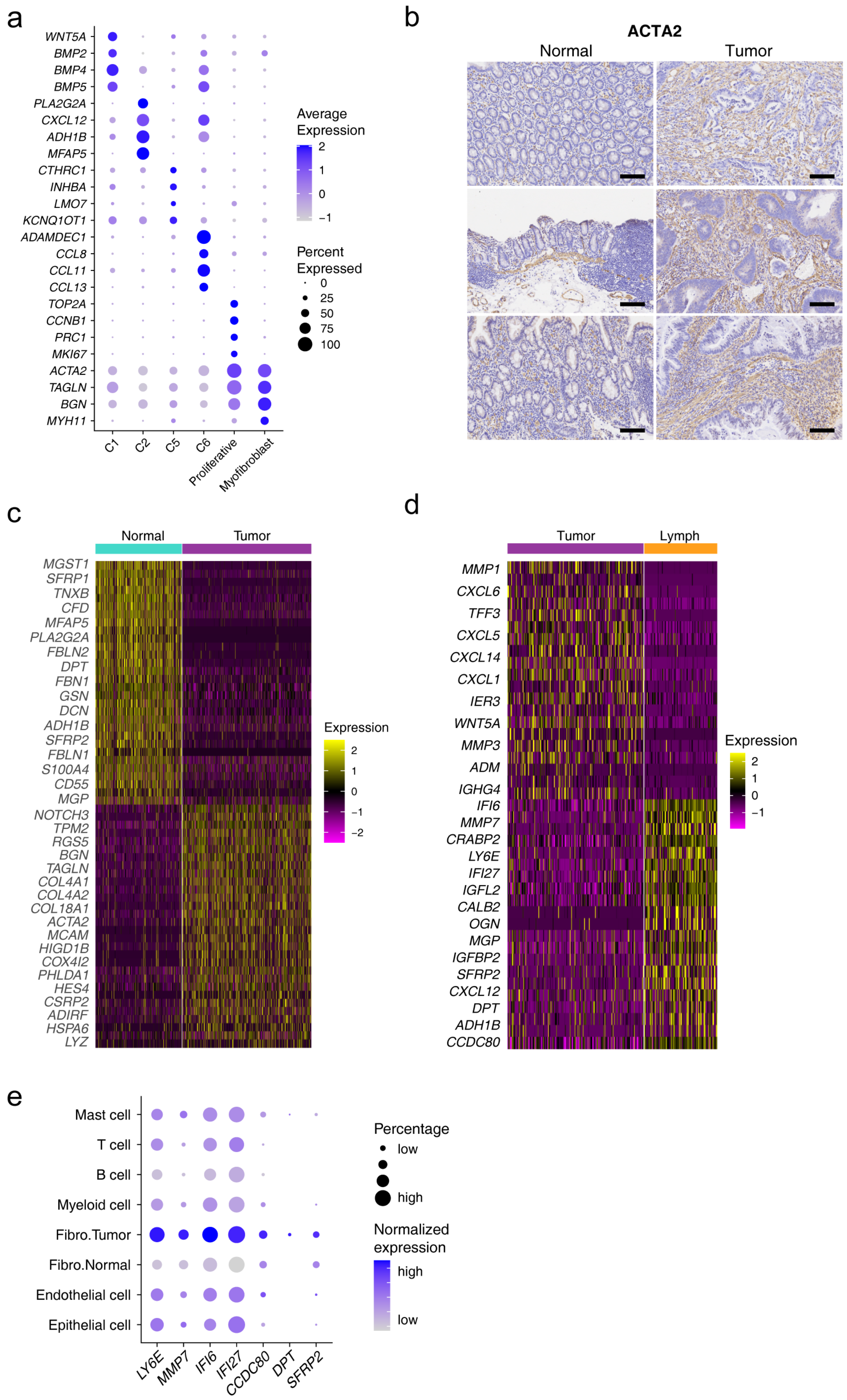

Supplementary Fig. S7 DEGs and identified markers of fibroblasts during SBA progression. a Dot plots presenting the normalized expression levels of corresponding markers of each fibroblast cluster in the 10× dataset. **b** Representative IHC staining of ACTA2 in adjacent normal and primary tumor tissues (original magnification 100×). The rows of the paired normal and tumor samples are from the same patients, and three individual patients are listed. Scale bar, 100 μm. **c** Heatmap showing DEGs of fibroblasts from tumor tissues compared with normal tissues in the 10× dataset. **d** Heatmap showing DEGs of fibroblasts from primary tumor tissues compared with lymph node tumor tissues in the STRT dataset. **e** Dot plots presenting the normalized expression levels of 8 increasingly expressing markers during tumor progression in the STRT dataset.

Supplementary Fig. S8

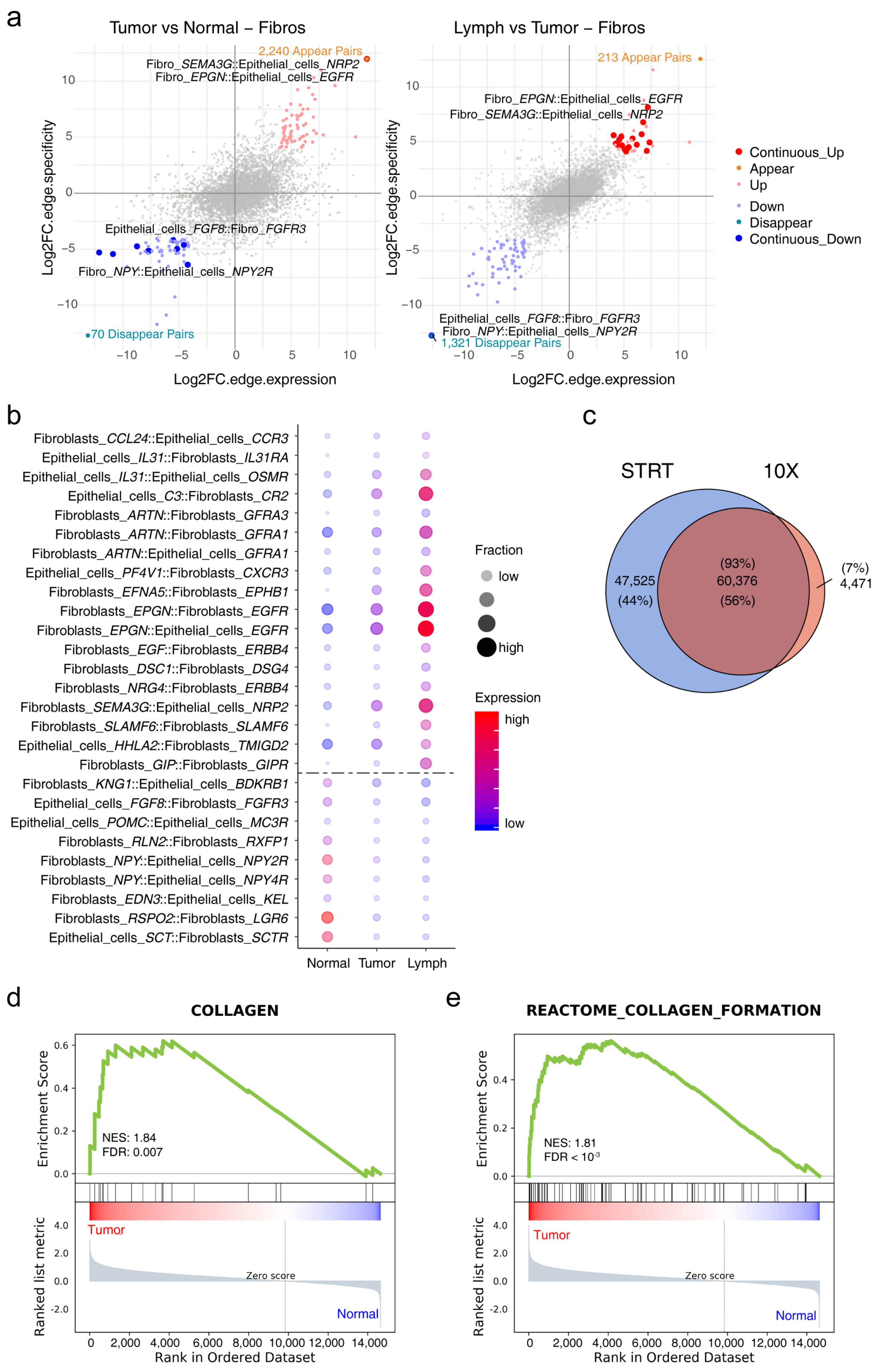

Supplementary Fig. S8 Different expression interactions of fibroblasts and epithelial cells during SBA progression. a Scatterplot showing differential expression interaction pairs of epithelial cells and fibroblasts during tumor progression. **b** Dot plot showing continuously differential expression of interaction pairs in different malignant cell and fibroblast pairs. **c** Venn plot showing interaction pairs of different types of the fibroblasts and malignant cells between the STRT and 10× datasets. **d** GSEA result of the collagen and reactome collagen formation gene sets enriched in fibroblasts from tumor tissues compared with normal tissues.

Supplementary Fig. S9

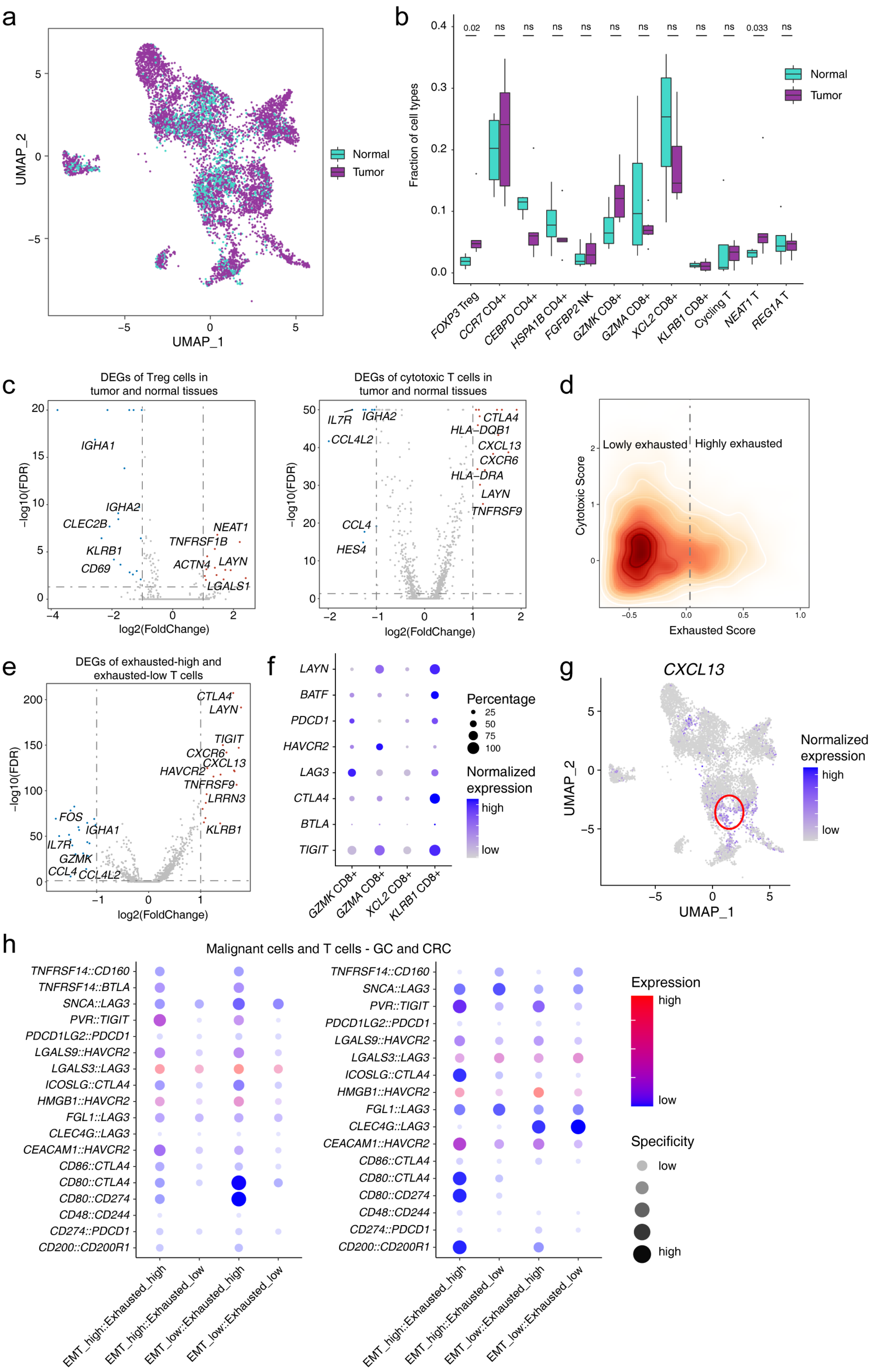

Supplementary Fig. S9 Analysis of T cell exhaustion in SBA. **a** UMAP plot exhibiting T cells from tumor and normal tissues in the 10× datasets. **b** Boxplot presenting cell percentage of different T cell subclusters between tumor and normal tissues in the 10× dataset, with "ns" representing no significance and *P* values calculated by *t*-test. **c** Volcano plot exhibiting DEGs of T cells between tumor and normal tissues in Treg cells and cytotoxic cells. **d** Density plot showing the separation of T cells with low and high T cell exhausted signatures in the 10× dataset. **e** Volcano plot showing DEGs of T cells with high and low exhausted signatures. **f** Dot plots presenting the normalized expression level of exhausted markers of $CD8^+$ T cells in the 10× dataset. **g** UMAP plot showing the expression of *CXCL13* in T cells from the 10× dataset. **h** Dot plot showing expression of interaction pairs in the malignant cells with low and high EMT signatures and T cell with low and high exhausted signatures of GC and CRC in responding 10× datasets. The used datasets are subsets of the GSE134520 and GSE132465 datasets in the GEO database.

Supplementary Fig. S10

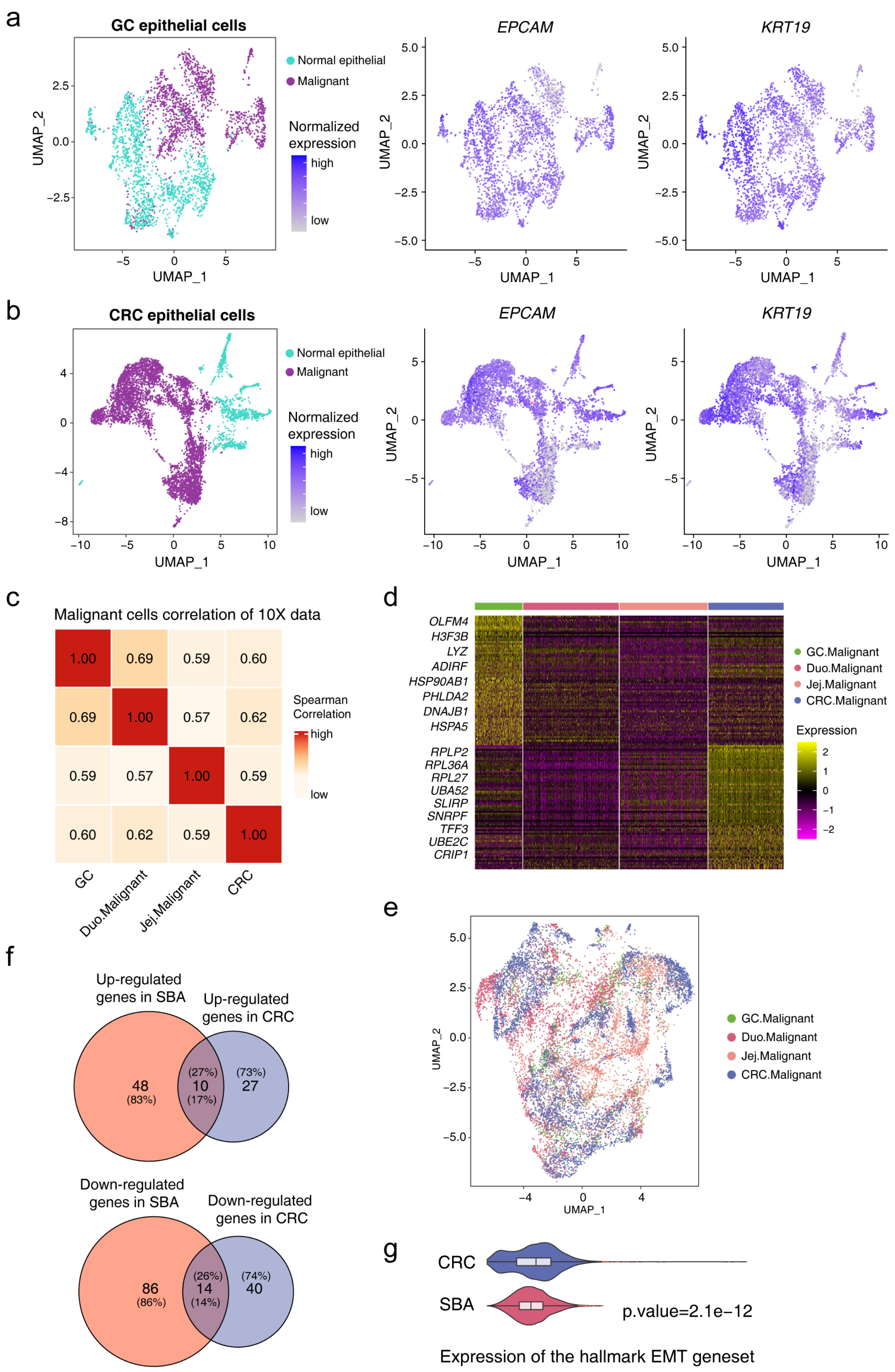

Supplementary Fig. S10 Analyses of gastrointestinal cancer datasets and comparison of gastrointestinal cancers. a, b Identification of malignant cells and normal epithelial cells and the expression of *EPCAM* and *KRT19* in the GC and CRC 10× datasets. The used datasets are subsets of the GSE134520 and GSE132465 datasets in the GEO database. **c** Heatmap showing Spearman correlation of malignant cells from different intestinal regions of SBA and other gastrointestinal cancers. **d** Heatmap showing the expression of DEGs of GC and CRC in all the malignant cells. **e** UMAP plot representing the integration of gastrointestinal cancers. **f** Venn plot showing interactions of markers of the malignant cells and normal epithelial cells between SBA and CRC in responding 10× datasets. **g** Violin plot showing expression of the hallmark EMT geneset between CRC and SBA in responding 10× datasets. The *P* value was calculated by *t*-test.

Supplementary Fig. S11

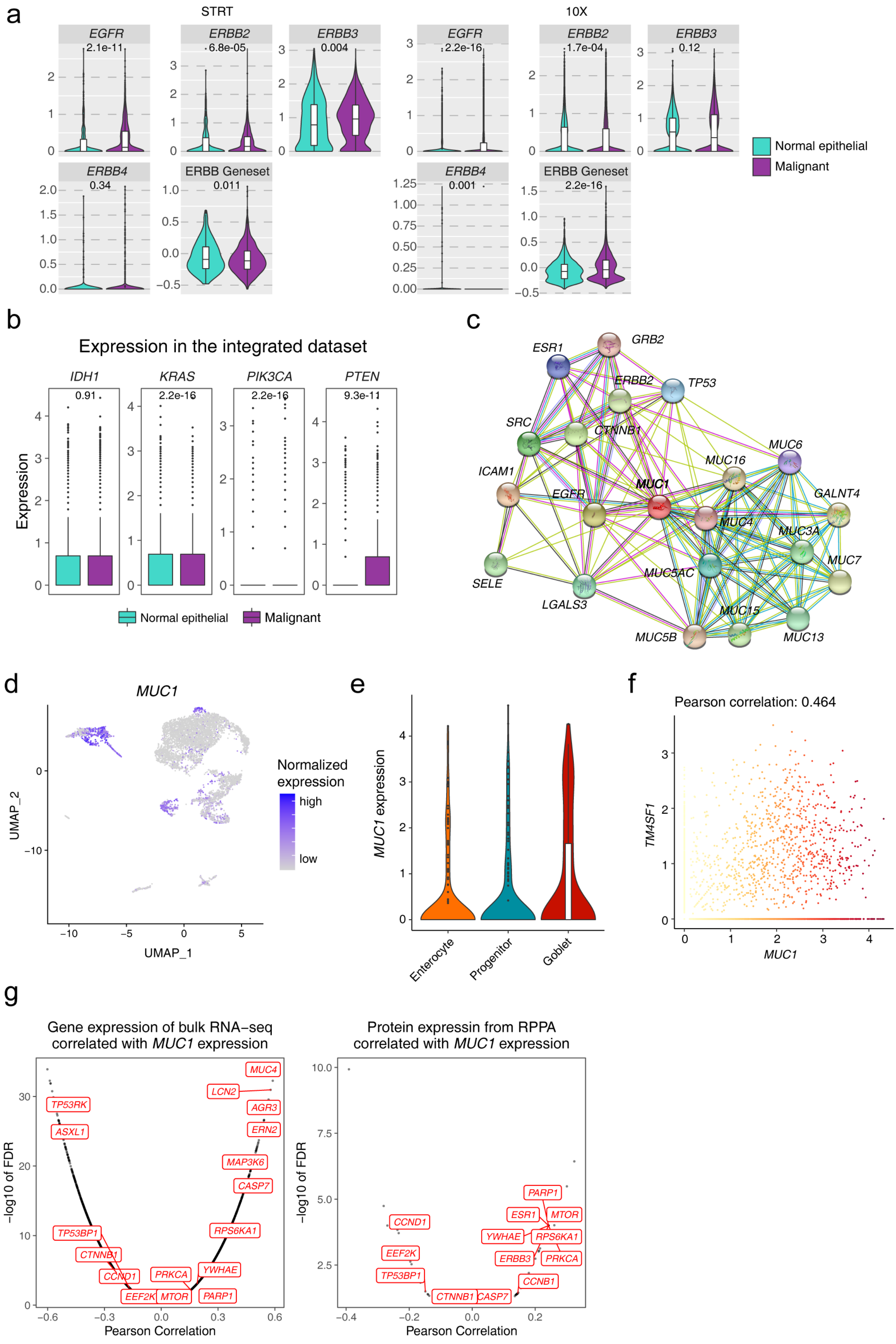

Supplementary Fig. S11 Expression of potential targets and *MUC1* as a malignant marker. **a** Violin plot exhibiting expression levels of key genes in the ERBB pathway and the ERBB family gene set score in the malignant cells and normal epithelial cells separately in the STRT and 10× datasets, with "ns" representing no significance and *P* values calculated by *t*-test. **b** Boxplot showing the expression of frequently-mutated genes of SBA in the integrated dataset, with "ns" representing no significance and *P* values calculated by *t*-test. **c** The protein-protein interaction network with the hub gene *MUC1* provided by the STRING database (version 11.5). **d** UMAP plot showing the expression of *MUC1* in normal epithelial cells of the 10× dataset. **e** Violin plot showing the expression of *MUC1* in the normal epithelial cells of the STRT dataset. **f** Scatterplot showing the Pearson correlation of *TM4SF1* with *MUC1* in the 10× dataset. **g** The first part of the scatterplot showing the Pearson correlation of the expression of *MUC1* and all the other genes according to the bulk RNA-seq of CRC. The second part of the scatterplot showing the Pearson correlation of the expression of *MUC1* and protein expression levels according to the RPPA data of CRC.

Supplementary Fig. S12

a

Datasets	Numbers of drugs (remained)	Published date
CTRP	538	2015
PRISM	1,492	2019
GDSC1	318	2016
GDSC2	173	2019

b

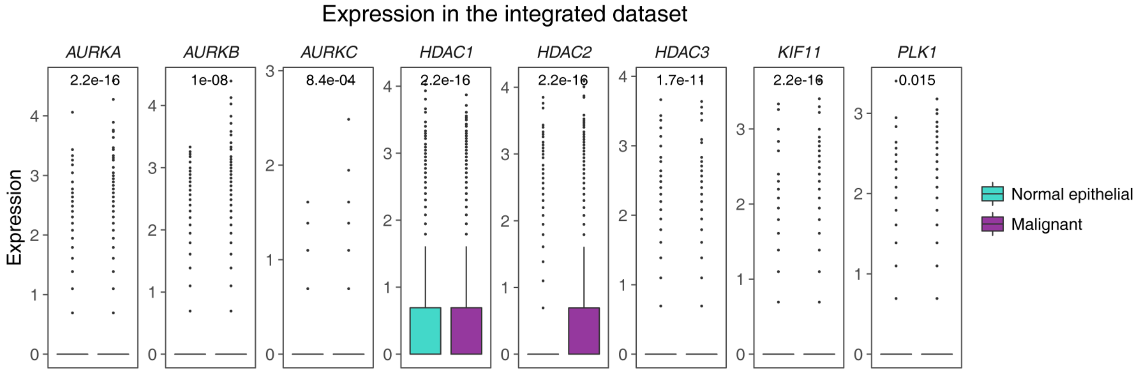

c

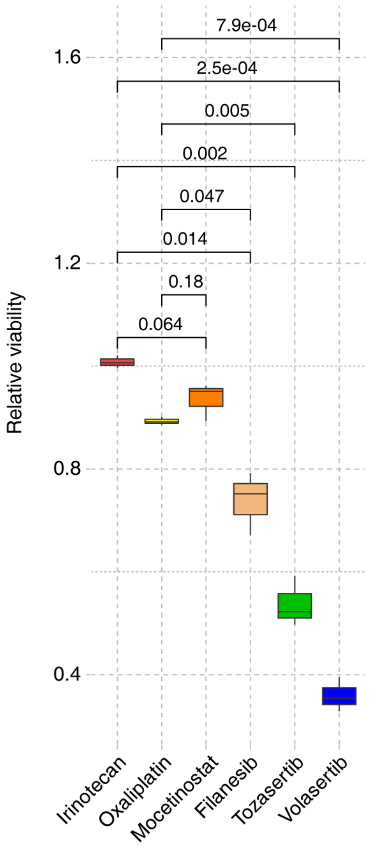

d

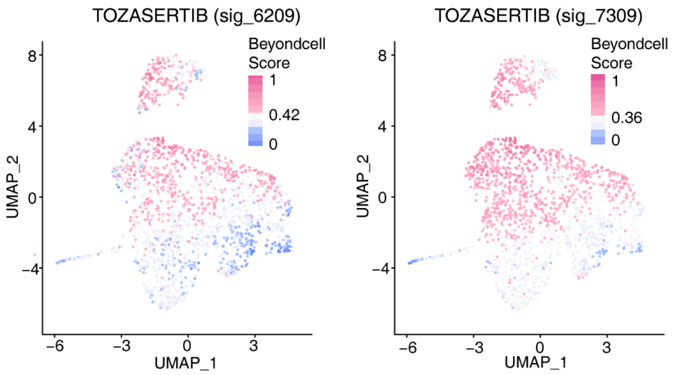

Supplementary Fig. S12 Analysis of predicted specific drugs and verification in SBA. **a** Information of used databases for predicting drugs. **b** Boxplot showing the expression of target genes of the four selected drugs in the integrated dataset, with “ns” representing no significance and *P* values calculated by *t*-test. **c** Boxplot showing the relative viability of selected drugs in the HUTU-80 cell line at the concentration of 0.1 μM, with “ns” representing no significance and *P* values calculated by *t*-test. **d** Evaluated drug sensitivity of tozasertib in single cells from the STRT dataset based on Beyondcell.
